# Supplementary material for: PAWS1 controls Wnt signalling through association with casein kinase 1α
Source: EMBO Rep. 2018 Mar 7;19(4):e44807. doi: 10.15252/embr.201744807 (PMC5891436; doi:10.15252/embr.201744807)
Supplement: Supplementary file 1 — Expanded View Figures PDF [file EMBR-19-e44807-s001.pdf]

Expanded View Figures

Figure EV1. Manipulation of PAWS1 in *Xenopus* embryos and human U2OS cells.

A–C Ectopic axis induction in *Xenopus* embryos following xPAWS1 mRNA injection. *Xenopus* embryos were injected at the one-cell stage with 500 pg of either HA_xPAWS1 (B) or xPAWS_HA mRNA(C). A variety of dorsalised phenotypes were observed including enlarged cement glands (asterisk), partial (arrowhead) and complete secondary axis (arrow). Scale bars are 2 mm.

D–I Dissociated animal caps injected with 50 pg of β-catenin_GFP mRNA were imaged over 3 h following treatment with the GSK3β inhibitor CHIR99021. Maximum intensity projection of β-catenin_GFP-injected cells before (D) and 3 h (E) after CHIR99021 treatment, demonstrating stabilisation and nuclear localisation of β-catenin_GFP in the absence of xPAWS1. Single z-section of a β-catenin_GFP expressing cell and corresponding fluorescence intensity profile across the nucleus before (F and G) and following 3 h of CHIR99021 treatment (H and I). Cells were imaged using a Zeiss LSM710 microscope, and intensity measurements from a single z-section were taken using Zen Black software. Scale bars are 20 μm.

J Expression level of Myc-tagged(MT)xPAWS1 and MTxPAWS1 mutants at stage 10. Extracts from embryos injected with 250 pg of MTxPAWS1 and MTxPAWS1 mutants were immunoblotted with antibodies against Myc-tag (green) and α-tubulin (red). The image was captured with a Li-Cor Odyssey scanner using Image Studio software (Li-Cor).

K Schematic illustration of the strategy employed to generate PAWS1-GFP knock-ins in U2OS cells. A pair of guide RNAs which recognise a genomic sequence upstream of the stop codon of PAWS1 gene was used in combination with a donor vector which inserts GFP in frame with the c-terminus of PAWS1.

L Cell extracts from $PAWS1^{GFP/GFP}$ cells compared with the $PAWS1^{-/-}$, confirmed that the gene in the reverse DNA strand of PAWS1, SLC5A10 is not disturbed.

M Mass fingerprinting analysis of PAWS1-GFP interactors from $PAWS1^{GFP/GFP}$-knock-in U2OS cells compared with $PAWS1^{-/-}$ U2OS cells (from Fig 5A) identified CK1α as a major interactor. The table shows total spectral counts for PAWS1 and CK1α tryptic peptides identified in anti-GFP IPs.

N The highlighted tryptic peptides identified by mass spectrometry on CK1α indicate the overall protein coverage. The included image was obtained using Scaffold V4.3 analysis of the LC-MS/MS data.

O Stable U2OS Flp-In Trex cells were subjected to 20 ng/ml doxycycline for inducing PAWS1-GFP expression or GFP expression alone for 24 h. Wnt3A or control medium was added to the cells for 6 h before lysis. 20 mg of cell extract was subjected to GFP-trap IP. Input (20 μg protein), 5% of the pull down and flow-through extract (20 μg protein) were subjected to SDS–PAGE followed by Western blot analysis with the indicated antibodies.

Source data are available online for this figure.

© 2018 The Authors

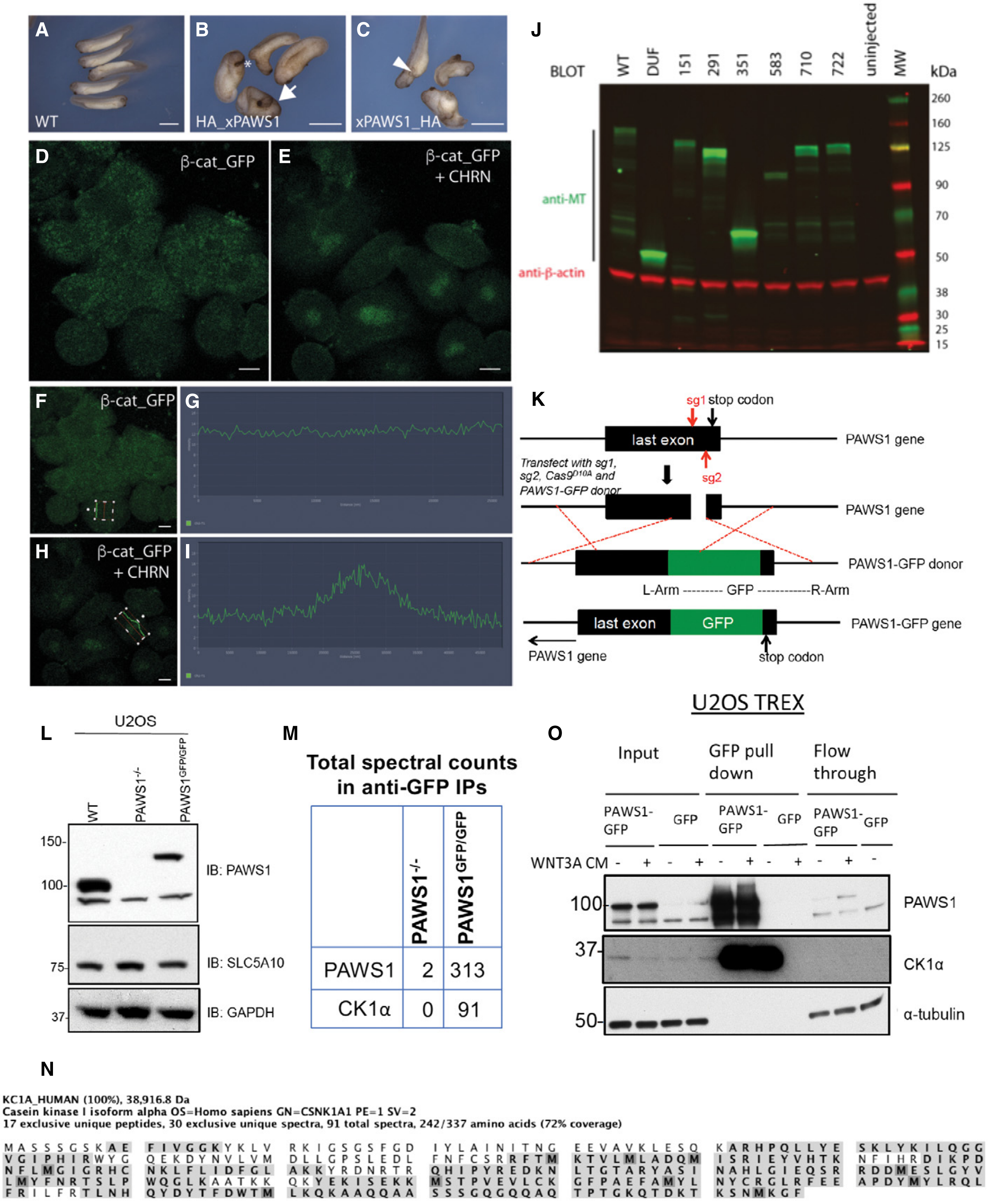

Figure EV1.

 © 2018 The Authors

Figure EV2. PAWS1 is phosphorylated at Ser^{614} by CK1α *in vitro*.

A ^{32}P autoradiography and Coomassie stain of SDS–PAGE gel after an *in vitro* kinase assay with GST-CK1α and GST-PAWS1-6xHis as a substrate.

B GST-PAWS1-6xHis phosphorylated by CK1α in A was excised from the gel, digested with trypsin and resolved by HPLC on a C_{18} column using increasing acetonitrile gradient. Analysis of the [$^{\gamma 32}P$] radioactivity peak at 54.1 min (P1) by LC-MS/MS revealed the phospho-peptide RPSVASSVSEEYFEVR.

C Analysis of the [$^{\gamma 32}P$] radioactivity peak P1 by LC-MS/MS revealed various phospho-peptides, of which RPSVASS(P)VSEEYFEVR was the only one to match the solid-phase Edman sequencing data. Together, they reveal that CK1α phosphorylates PAWS1 at Ser^{614}.

D ^{32}P autoradiography and Coomassie stain of SDS–PAGE after an *in vitro* kinase assay with $CK1\alpha^{WT}$ or $CK1\alpha^{KD}$ (kinase dead) and $PAWS1^{WT}$ or $PAWS1^{S614A}$ as substrates.

E Human $PAWS1^{WT}$, $hPAWS1^{S610A}$ and $hPAWS1^{S610A/S614A}$ induce axis duplication in *Xenopus* embryos.

© 2018 The Authors

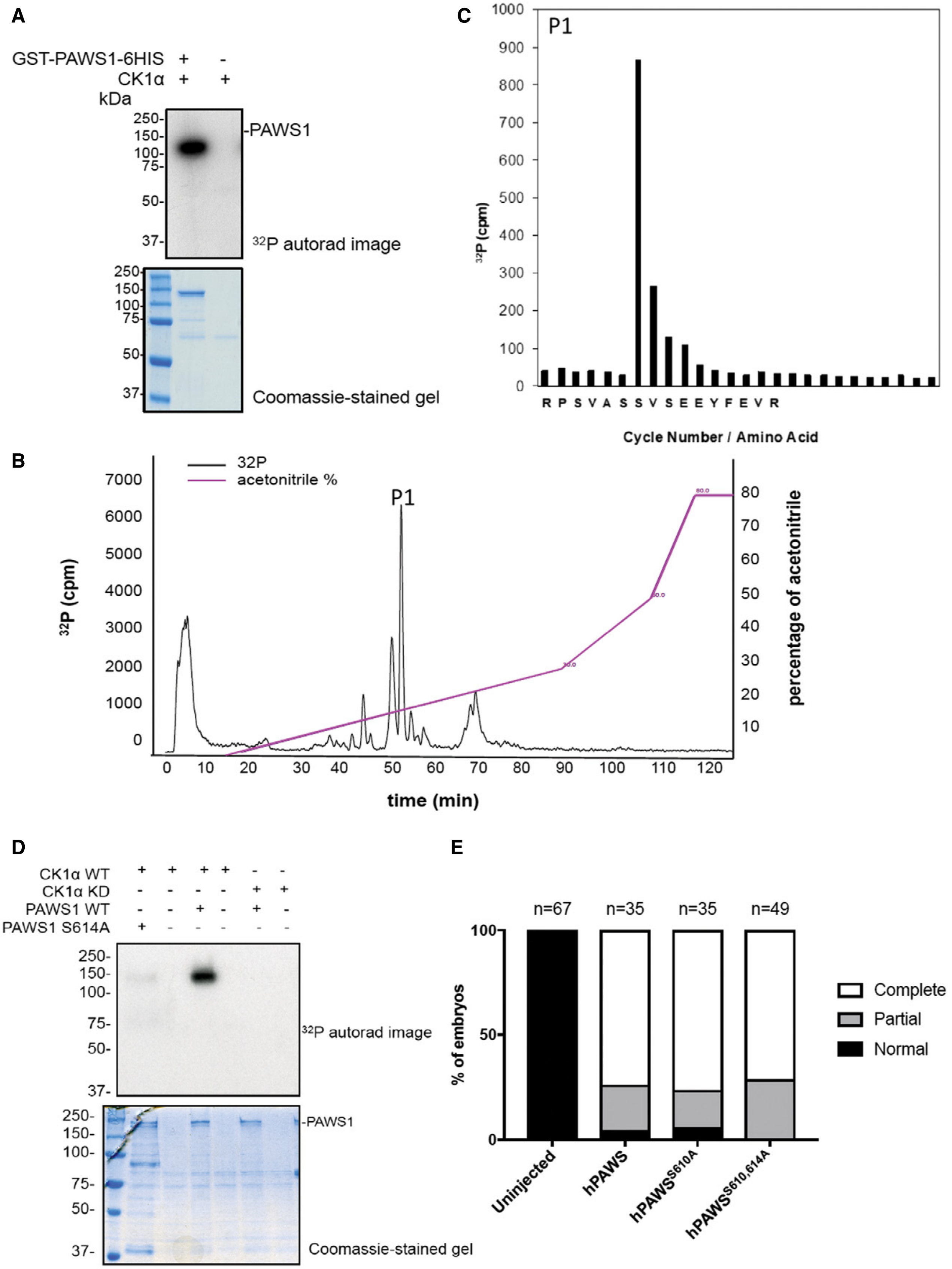

Figure EV2.

 © 2018 The Authors

Figure EV3. PAWS1 regulates the CK1α protein but not mRNA levels in cells.

A PAWS1^{WT} and PAWS1$^{-/-}$ U2OS cells were treated with 10 μM of the proteasome inhibitor Bortezomib for 6 h and extracts were analysed by Western blotting with the indicated antibodies.

B PAWS1^{WT} and PAWS1$^{-/-}$ U2OS cells were treated with 50 μM Bafilomycin A1 (BafA1) for 2 or 16 h, and extracts were analysed by Western blotting with the indicated antibodies.

C Correlation plots of PAWS1 and CK1α protein expression from Fig 6E based on densitometry (using ImageJ) quantification of immunoblots normalised to GAPDH levels (a.u.: arbitrary units). Each dot represents a cell line. Pearson r coefficient and one-tailed *P*-values were calculated with Prism6 software.

D As in (C), except that PAWS1 and CK1ε protein levels were quantified and plotted.

E CK1α mRNA in PAWS1$^{-/-}$ cells rescued with PAWS1^{WT} or GFP control analysed by quantitative RT–PCR. Data are represented as fold induction over controls and normalised internally to β-actin control. Error bars represent ± SEM (n.s.: no statistical significance; n = 3).

F Relative expression of PAWS1 and CK1α transcripts in the indicated cancer cell lines was measured by RT–PCR (n = 7; error bars represent ± SEM).

G Correlation plot of relative PAWS1 and CK1α mRNA expression (normalised to β-actin) in a subset of the indicated cancer cell lines. Pearson r coefficient and one-tailed *P*-values were calculated with Prism6 software.

Source data are available online for this figure.

© 2018 The Authors

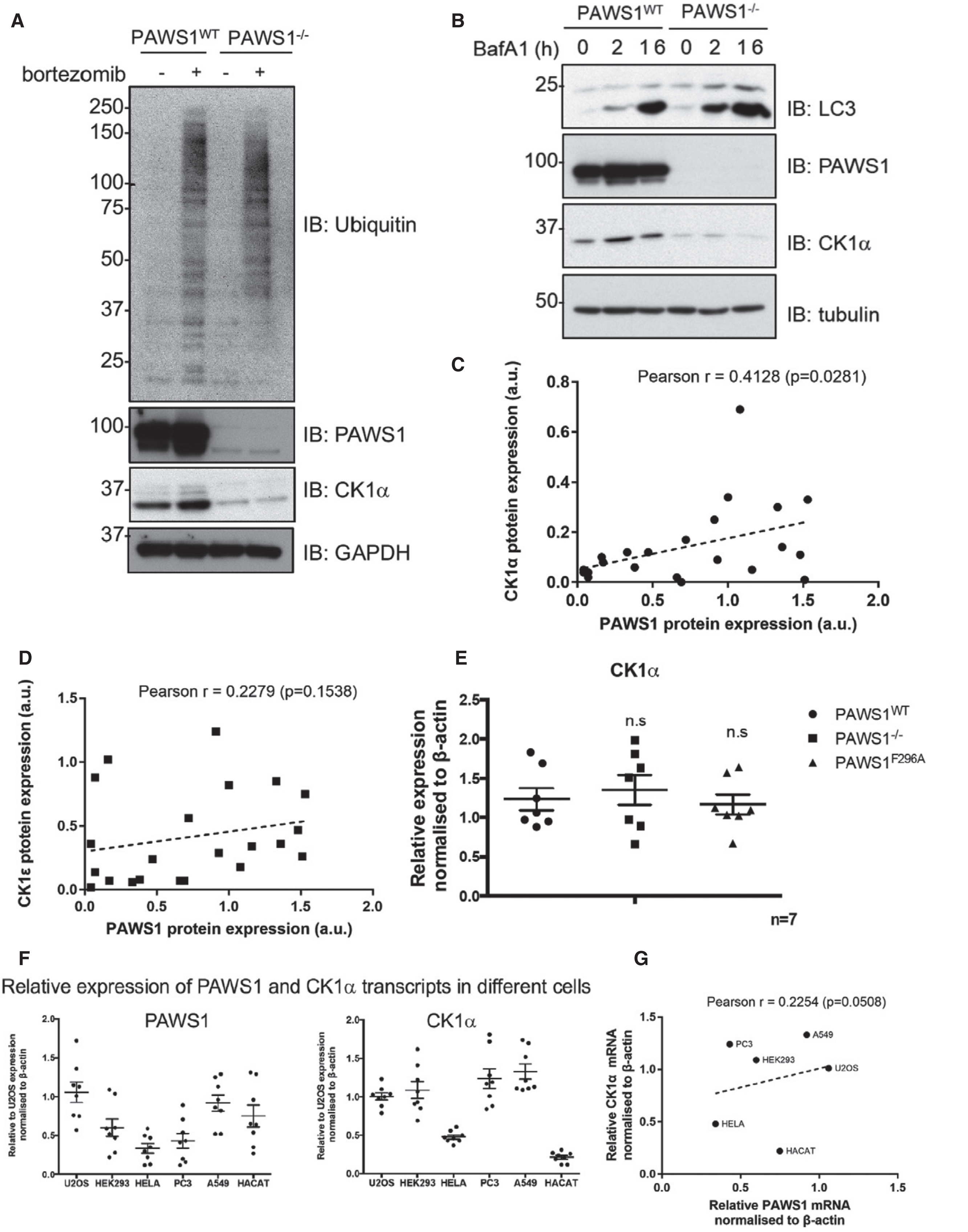

Figure EV3.

 © 2018 The Authors

Figure EV4. xPAWS1 does not induce nuclear localisation of NFAT.

A Dissociated animal cap cells injected with either 500 pg of NFAT_GFP or 500 pg of NFAT_GFP and 250 pg of xPAWS1_mCherrymRNA. Images are maximum intensity projections, scale bars = 50 μm.

B Nuclear localisation of NFAT_GFP was assessed before and after treatment with 0.025 μg/ml calcium ionophore. ns: no statistical significance (n = 4; error bars represent ± SD; Mann–Whitney test, unpaired, two-tailed with unequal variance, P = 0.6857).

C Expression of a catalytically inactive CK1α leads to Ca^{2+}-independent nuclear localisation of NFAT_GFP. Dissociated animal cap cells injected with either 500 pg of NFAT_GFP alone and 250 pg of H2B_RFP (top panels), 500 pg of NFAT_GFP, 250 pg H2B_RFP and 250 pg of MTxPAWS1 (middle panels), or 500 pg NFAT_GFP, 250 pg H2B_RFP, and 300 pg CK1α KD (bottom panels). Images represent a single z-frame at the level of the nucleus. Cells were imaged using a Zeiss LSM710 confocal microscope controlled with Zen Black software. Image analysis was performed using Zen blue software. Scale bars = 50 μm.

Source data are available online for this figure.

© 2018 The Authors

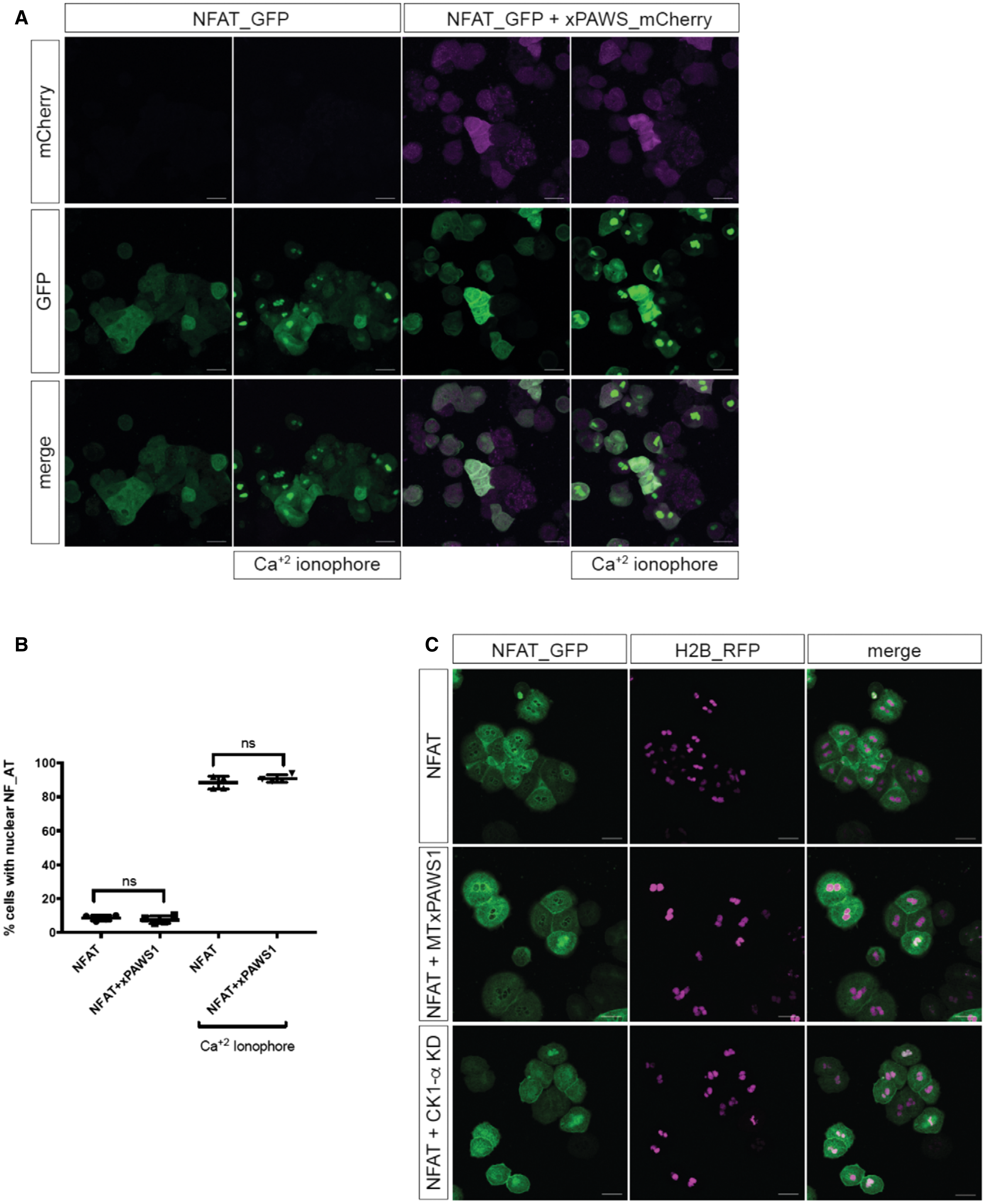

Figure EV4.

 © 2018 The Authors

Figure EV5. Effects of PAWS1 on Wnt pathway components.

A U2OS wild-type (WT) and PAWS1$^{-/-}$ (KO) cells were treated with control-conditioned medium or Wnt3A-conditioned medium and lysed at the indicated time points. Extracts (0.5 mg protein) were subjected to IPs using anti-CK1α or pre-immune IgG control (10 μg antibodies coupled to 10 μl packed protein-G sepharose beads). IPs were resolved by SDS–PAGE and immunoblotted with the indicated antibodies.

B Stable U2OS Flp-In TRex cells were treated with 20 ng/ml doxycycline for 16 h, for induction of PAWS1-GFP or GFP protein expression, and with Wnt3A conditioned medium or control medium for 3 h prior to lysis. GFP pull downs were resolved by SDS–PAGE and the gel was stained with Coomassie. Each lane was cut into five pieces, which were subsequently processed for protein identification by mass fingerprinting analysis.

C PAWS1-GFP interacting proteins were plotted using total spectral counts for selected individual protein for both control (filled) and Wnt3A (open) conditions. Total spectral counts are defined as the sum of all the spectra associated with a specific protein within a sample, which includes also those spectra that are shared with other proteins. A spectral count of 3 or more in either control or Wnt3A condition in PAWS1-GFP IPs and no spectral counts in GFP control IPs were set as threshold for inclusion. All proteins, except those indicated with asterisks, were identified as endogenous PAWS1$^{GFP/GFP}$ interactors as well (Fig 5A).

D PAWS1 phospho-residues from gel slices 2 and 7 from (B) were identified by mass spectrometry. Residues denoted by "or" indicate that a specific single phospho-residue on the corresponding tryptic peptide could not be assigned but could be any one of those indicated.

E U2OS wild-type (WT) and PAWS1$^{-/-}$ cells were treated with control (L-CM) or Wnt3A conditioned medium (L3-CM) for 3 h and fractionated into cytoplasmic (C), nuclear (N), membrane (M) and cytoskeletal (Cs) fractions. Extracts (20 μg protein) from each fraction was resolved by SDS–PAGE, transferred onto PVDF membranes, which were probed by Western blotting with the indicated antibodies. α-Tubulin was used as a cytosolic marker, Lamin A/C as a nuclear marker, LRP6 and EGFR were used as membrane markers, and Vimentin was used as a cytoskeletal marker.

Source data are available online for this figure.

© 2018 The Authors

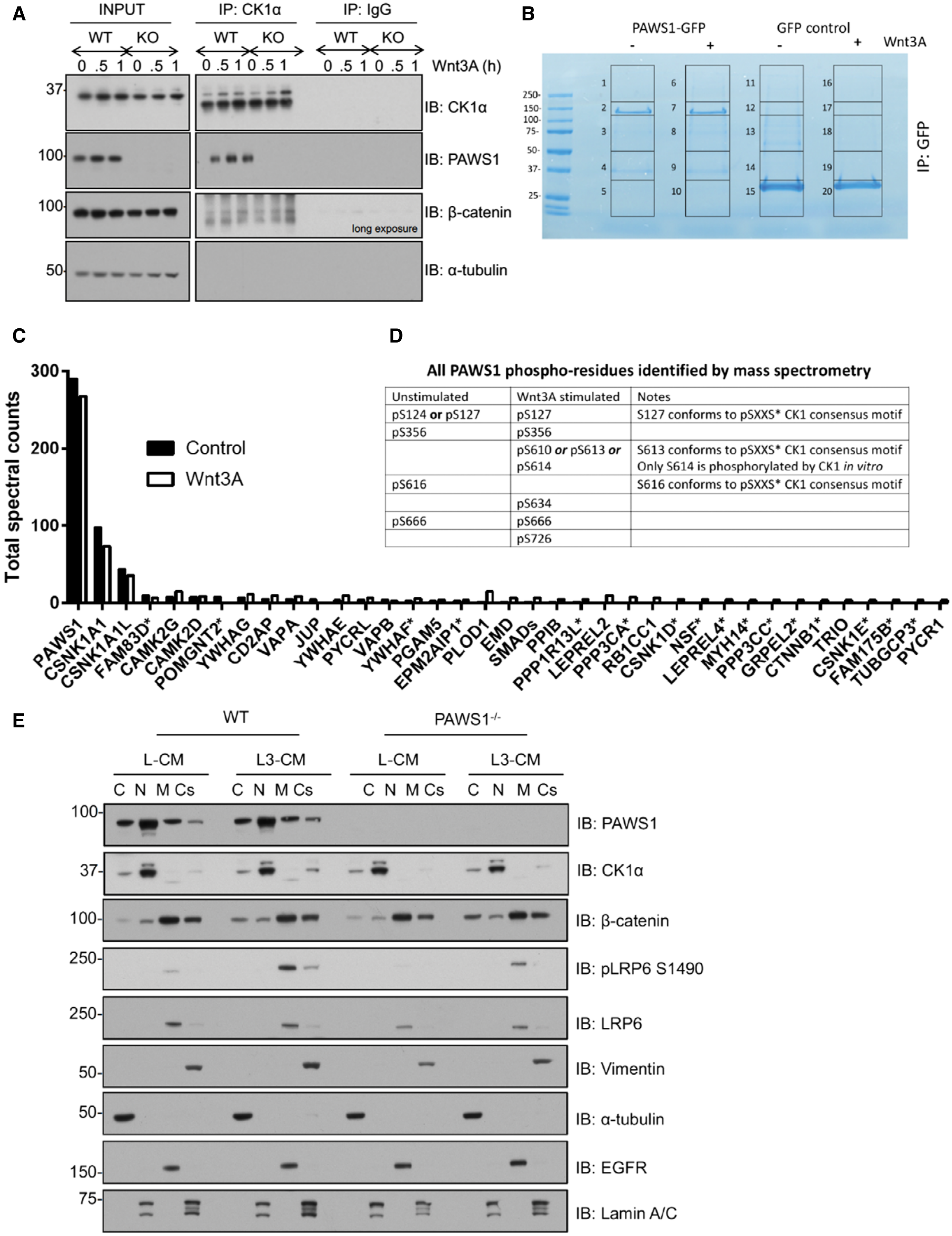

Figure EV5.

 © 2018 The Authors
