# Supplementary figures and images for: PAWS1 controls Wnt signalling through association with casein kinase 1α
Source: EMBO Rep. 2018 Mar 7;19(4):e44807. doi: 10.15252/embr.201744807 (PMC5891436; doi:10.15252/embr.201744807)

Fig EV1

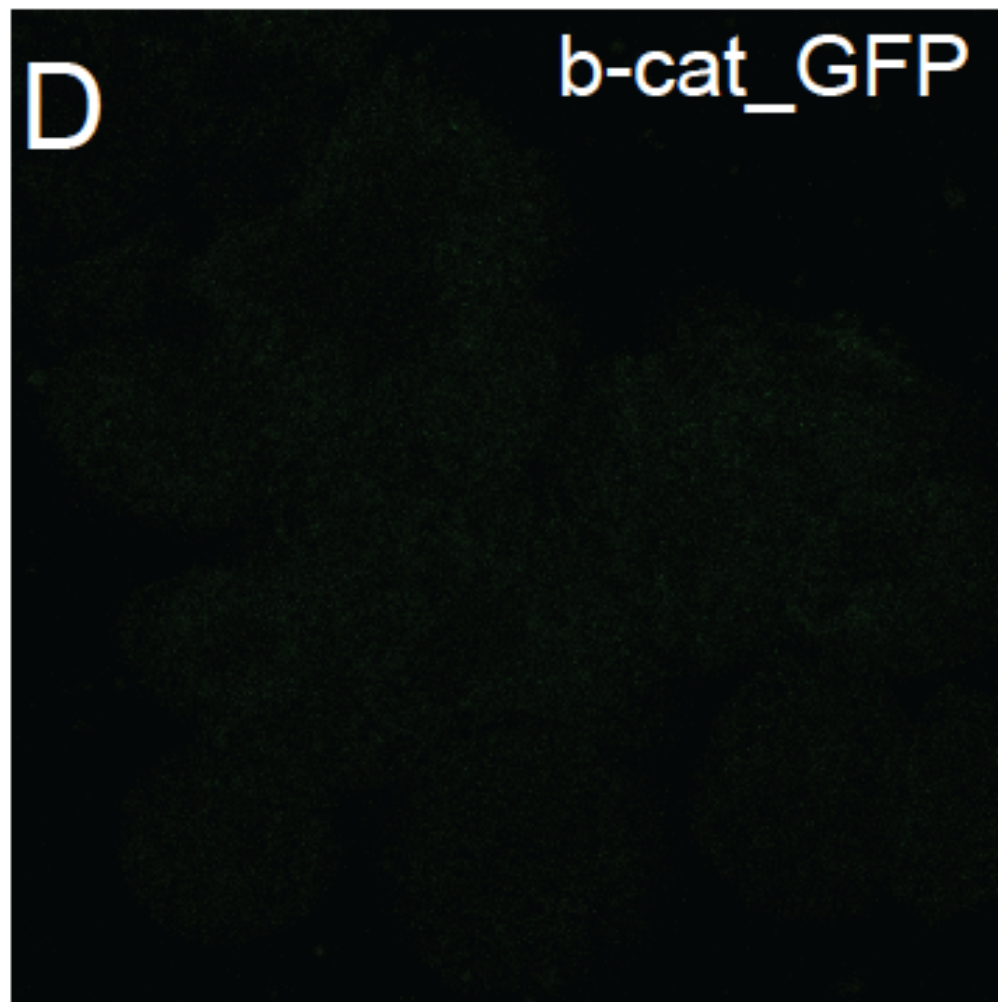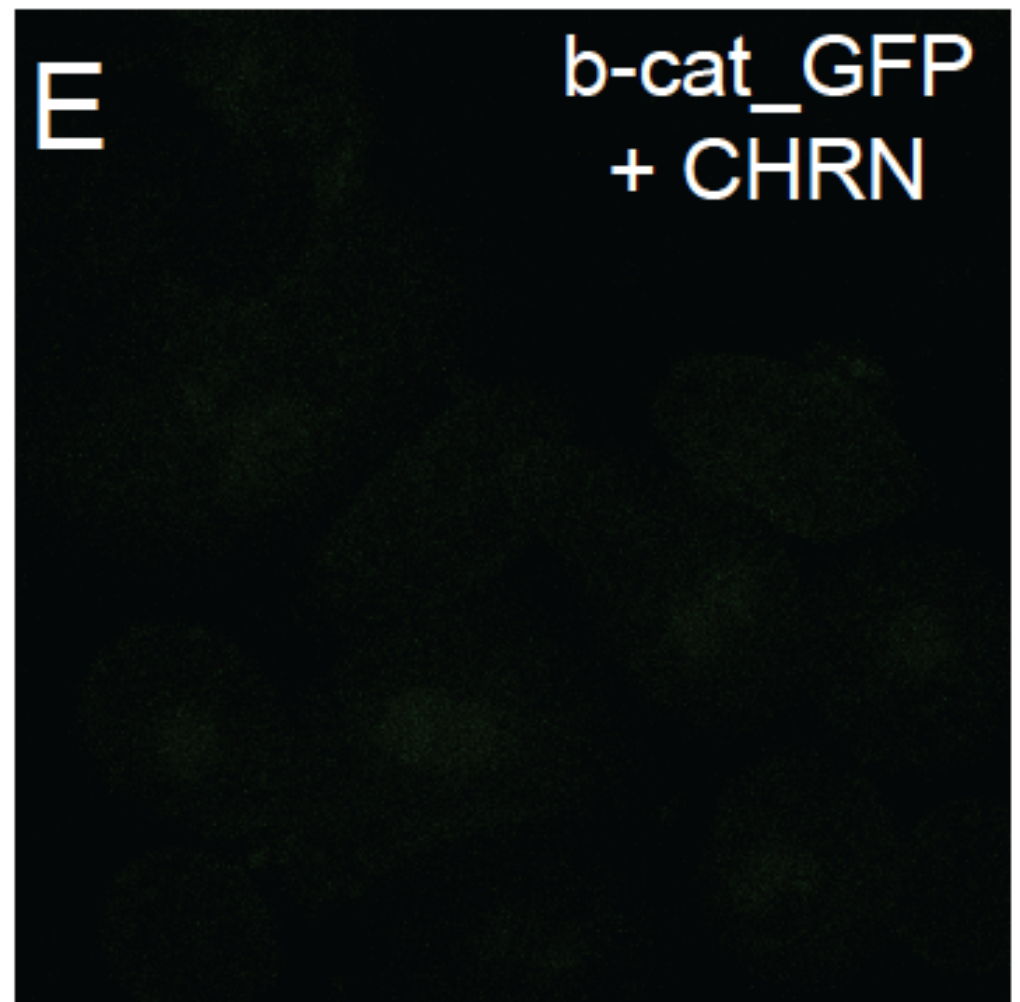

**L**

O

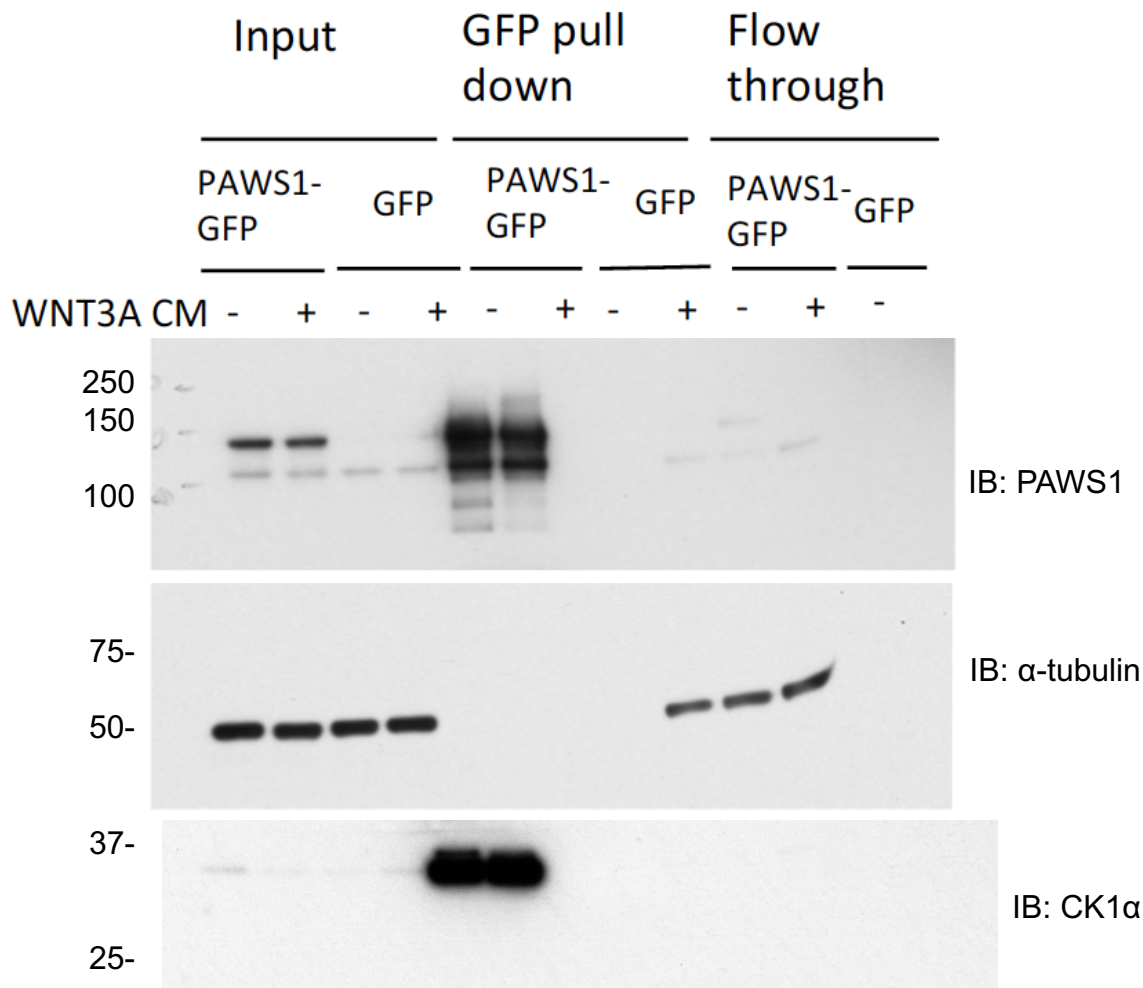

Supplement: Supplementary file 2 — Source Data for Expanded View [file EMBR-19-e44807-s009.zip › 44807_Source_Data_for_EV_Figures/Source_File_Figure_EV1.pdf]

Figure EV3

A

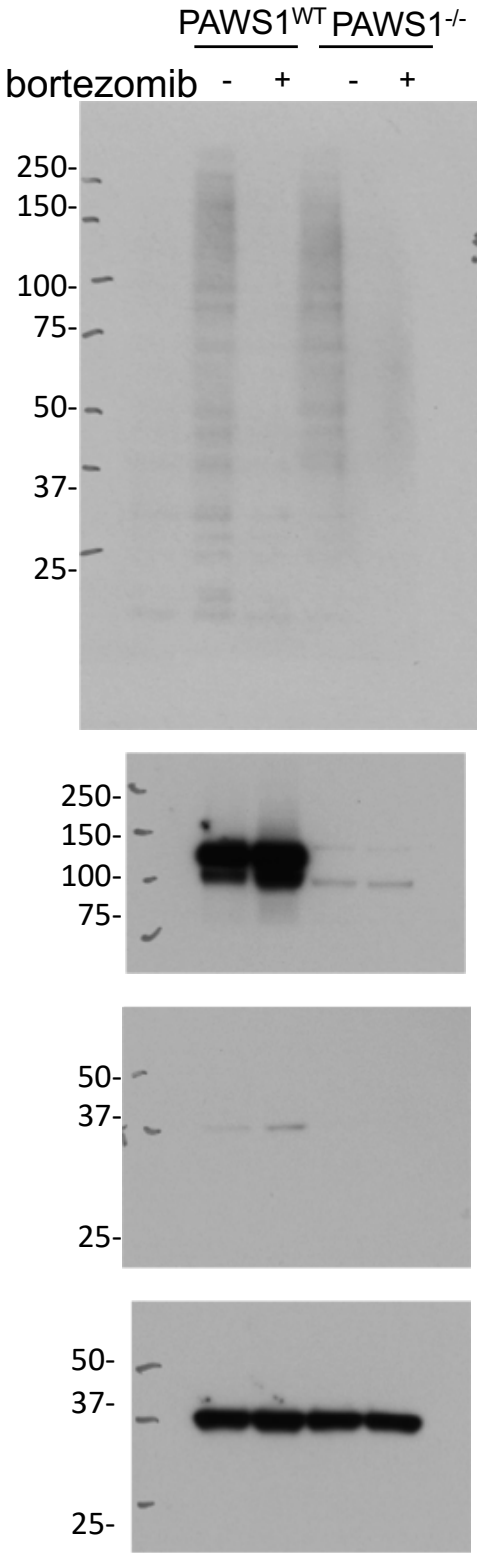

B

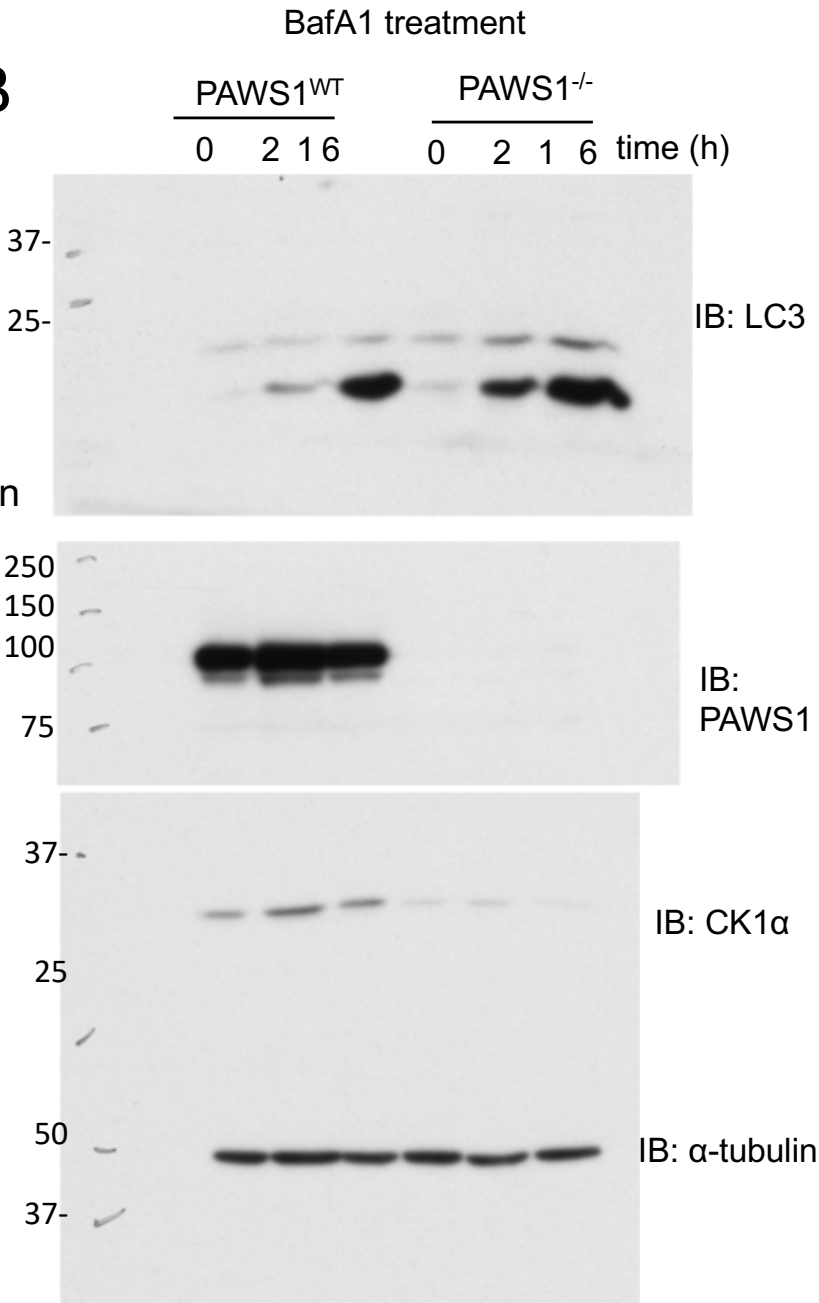

Supplement: Supplementary file 2 — Source Data for Expanded View [file EMBR-19-e44807-s009.zip › 44807_Source_Data_for_EV_Figures/Source_File_Figure_EV3.pdf]

Fig EV4A

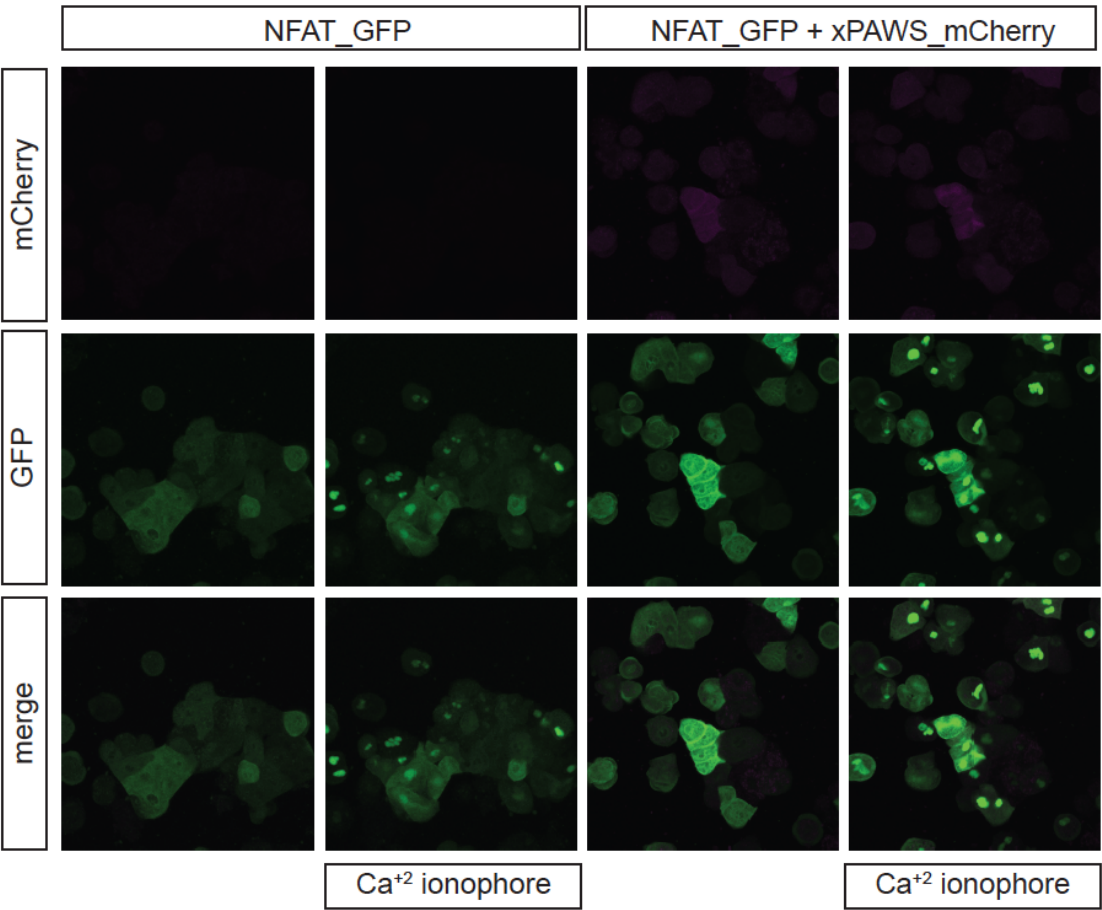

Fig EV4C

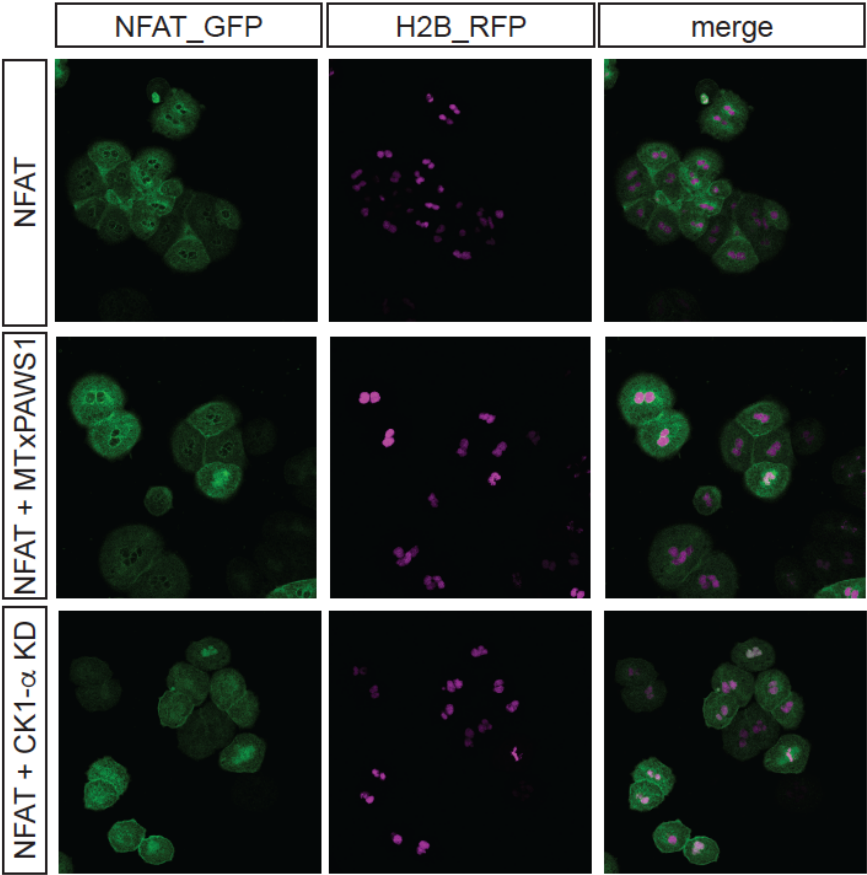

Supplement: Supplementary file 2 — Source Data for Expanded View [file EMBR-19-e44807-s009.zip › 44807_Source_Data_for_EV_Figures/Source_File_Figure_EV4.pdf]

Figure EV5A

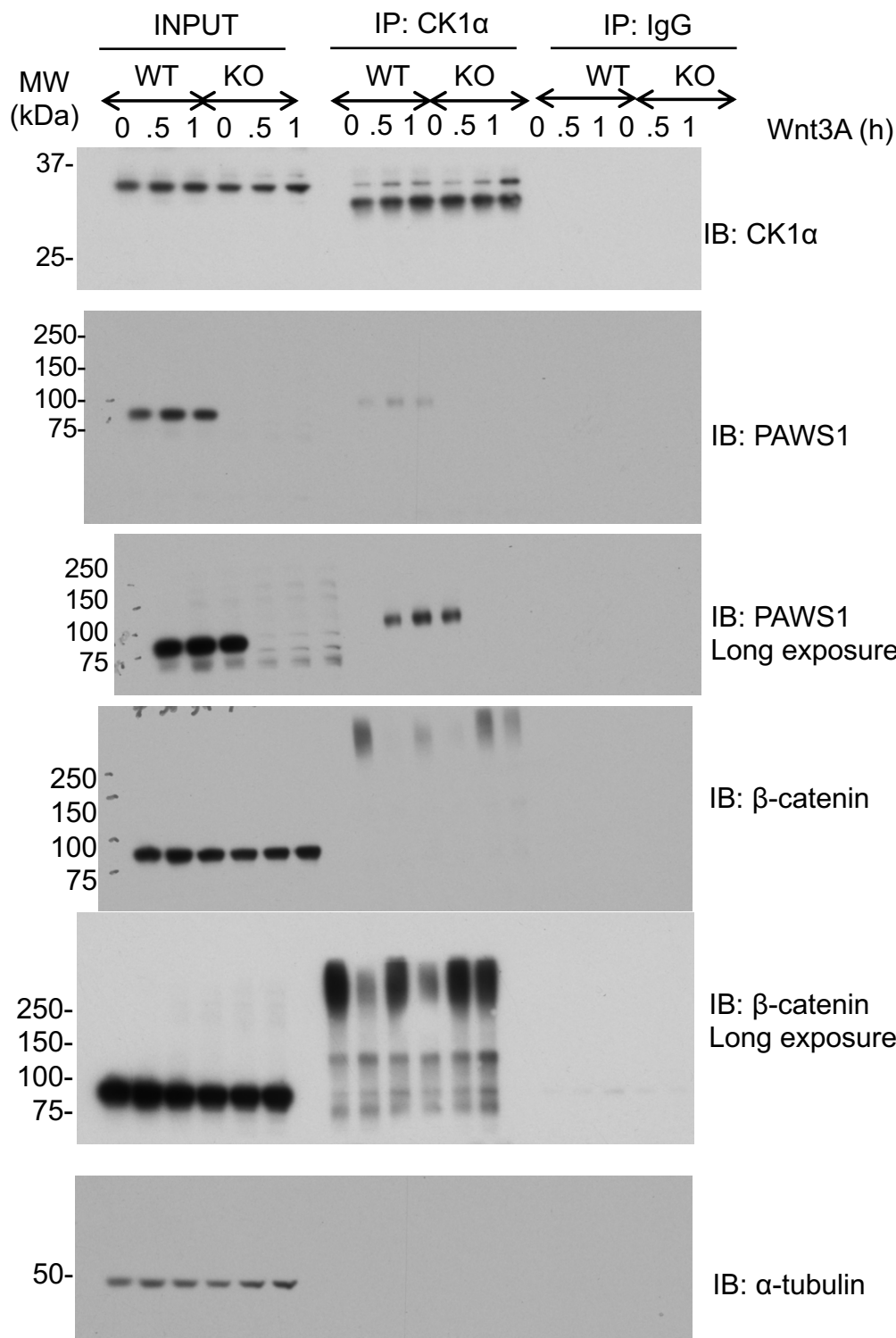

Figure EV5E

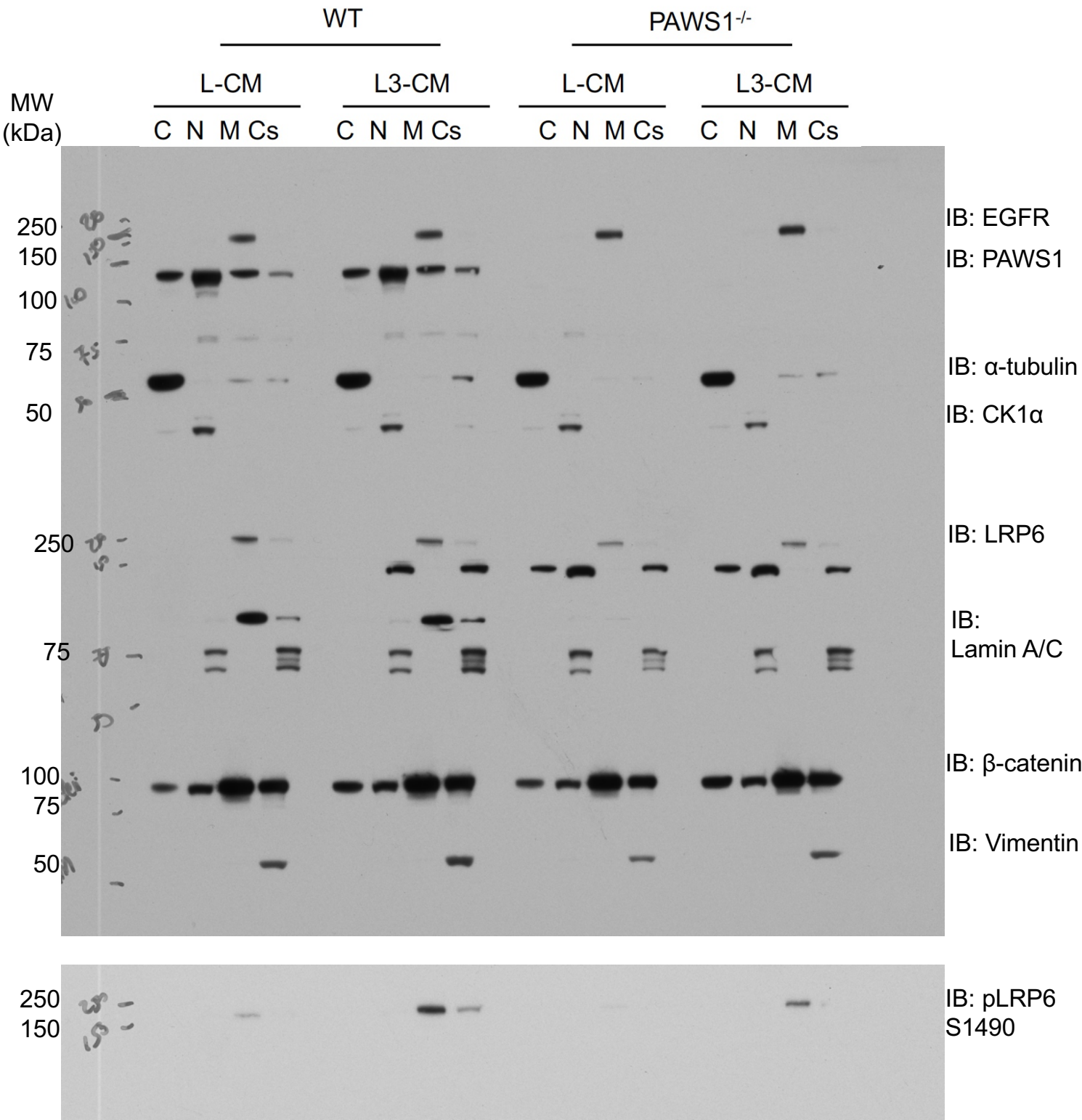

Supplement: Supplementary file 2 — Source Data for Expanded View [file EMBR-19-e44807-s009.zip › 44807_Source_Data_for_EV_Figures/Source_File_Figure_EV5.pdf]

Fig 2A

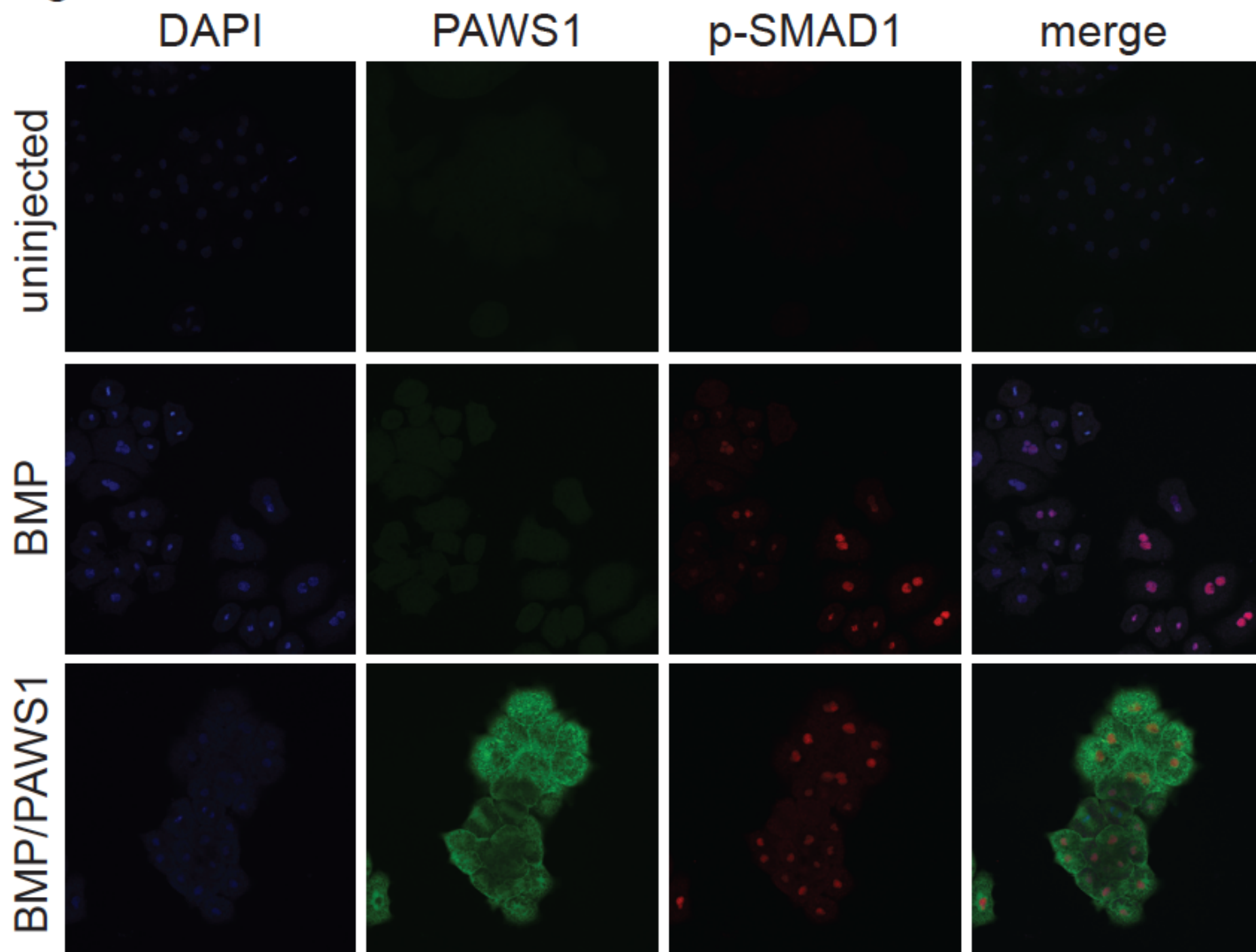

Figure 2C

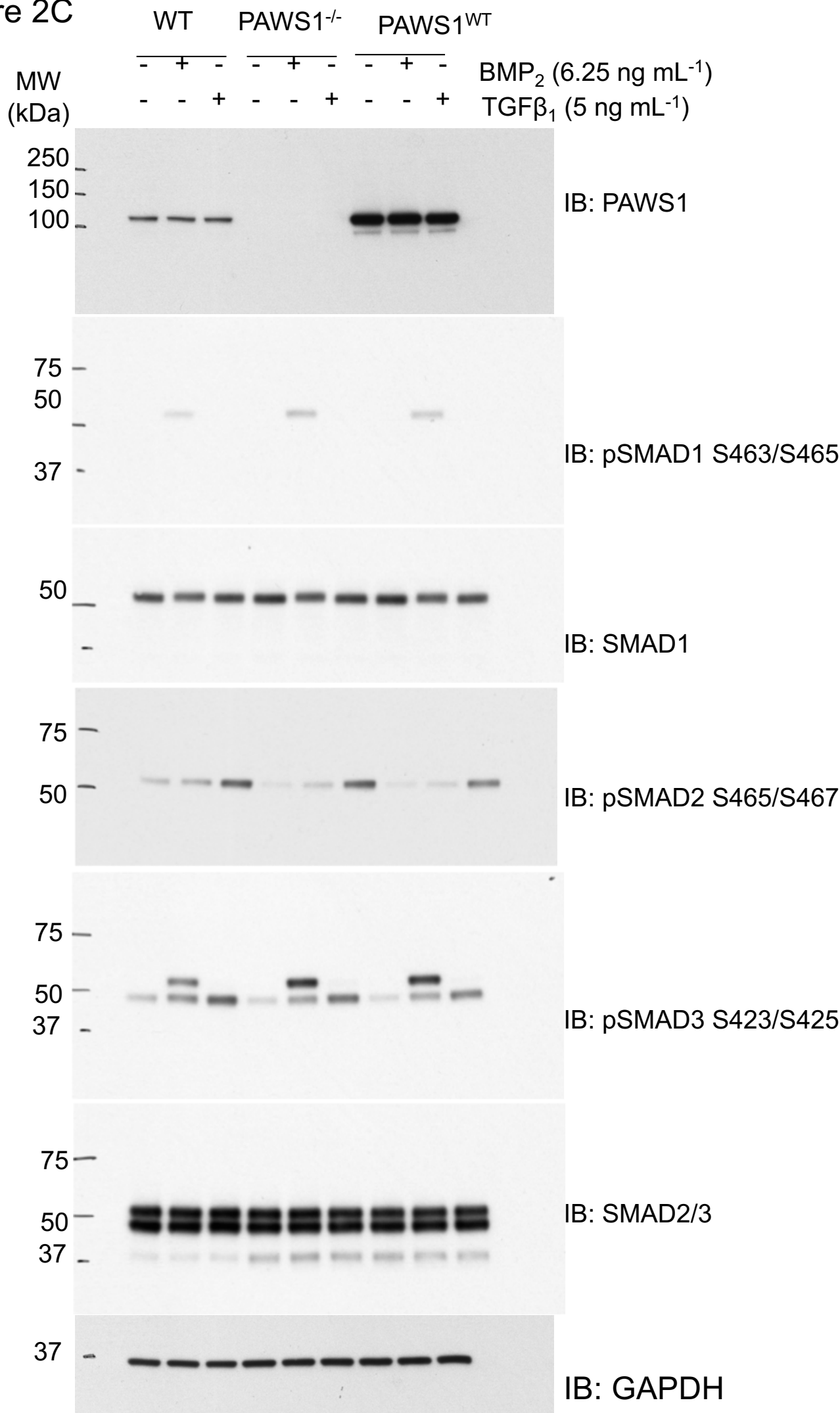

Supplement: Supplementary file 4 — Source Data for Figure 2 [file EMBR-19-e44807-s002.pdf]

Figure 5B

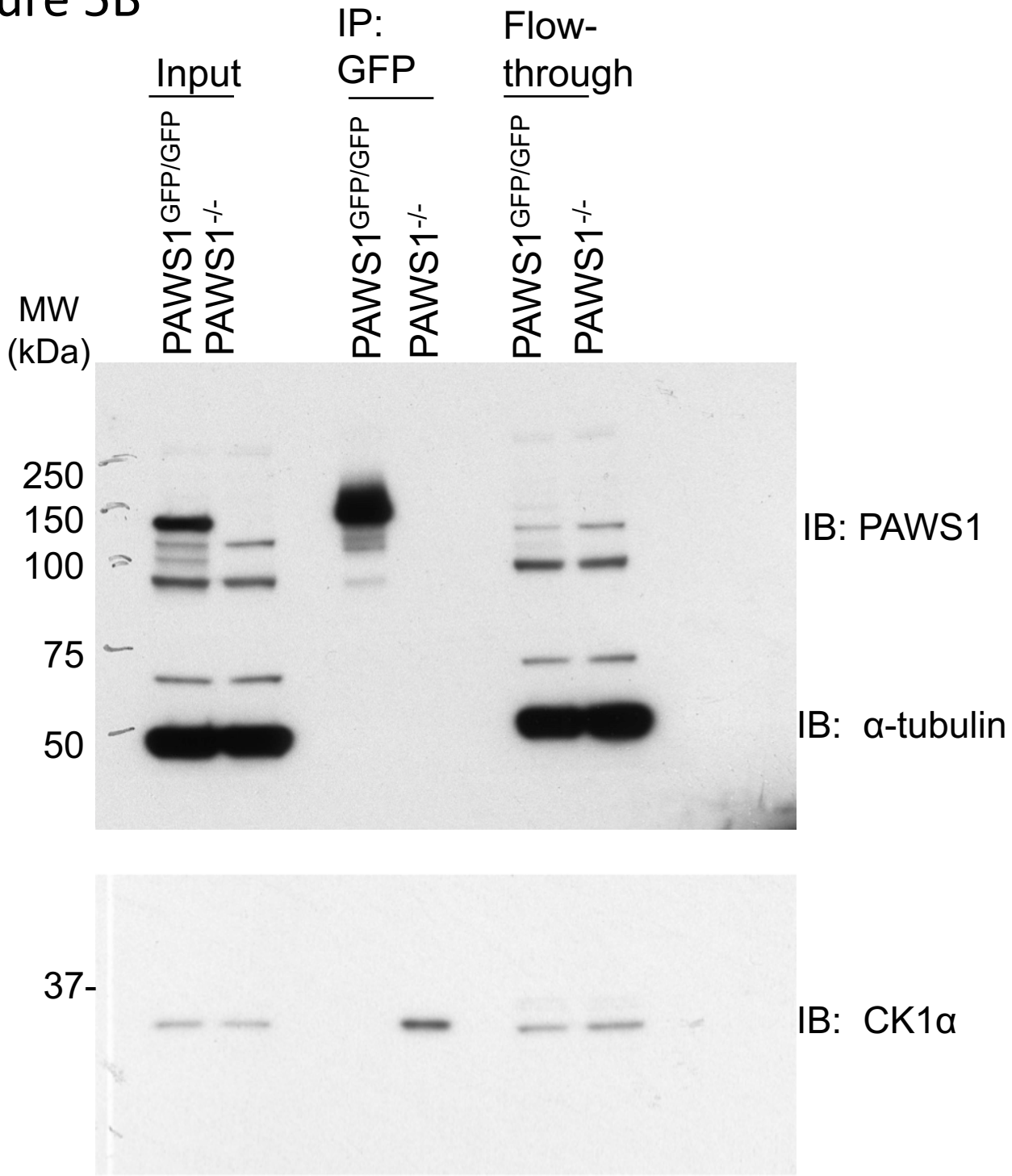

Figure 5C

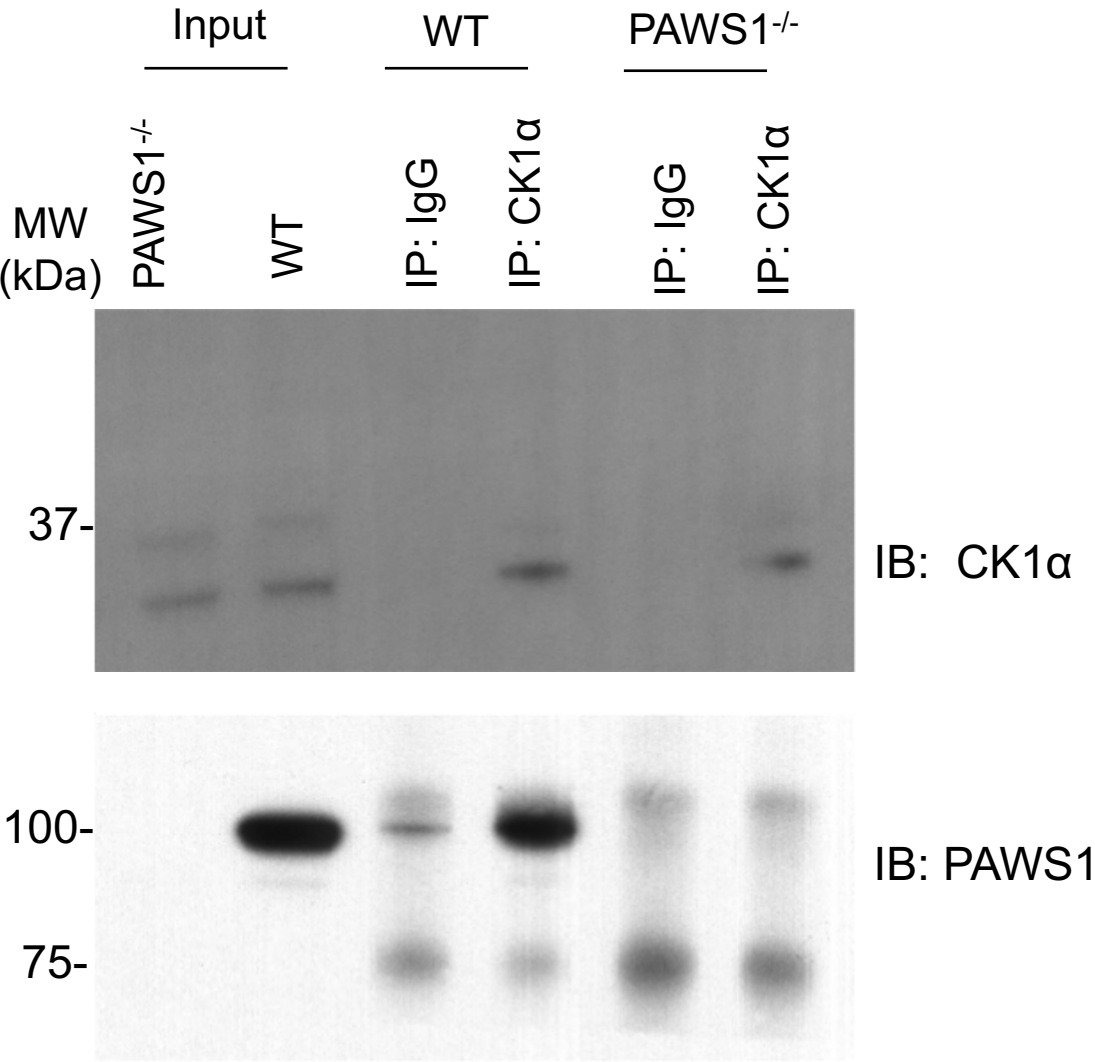

Figure 5D

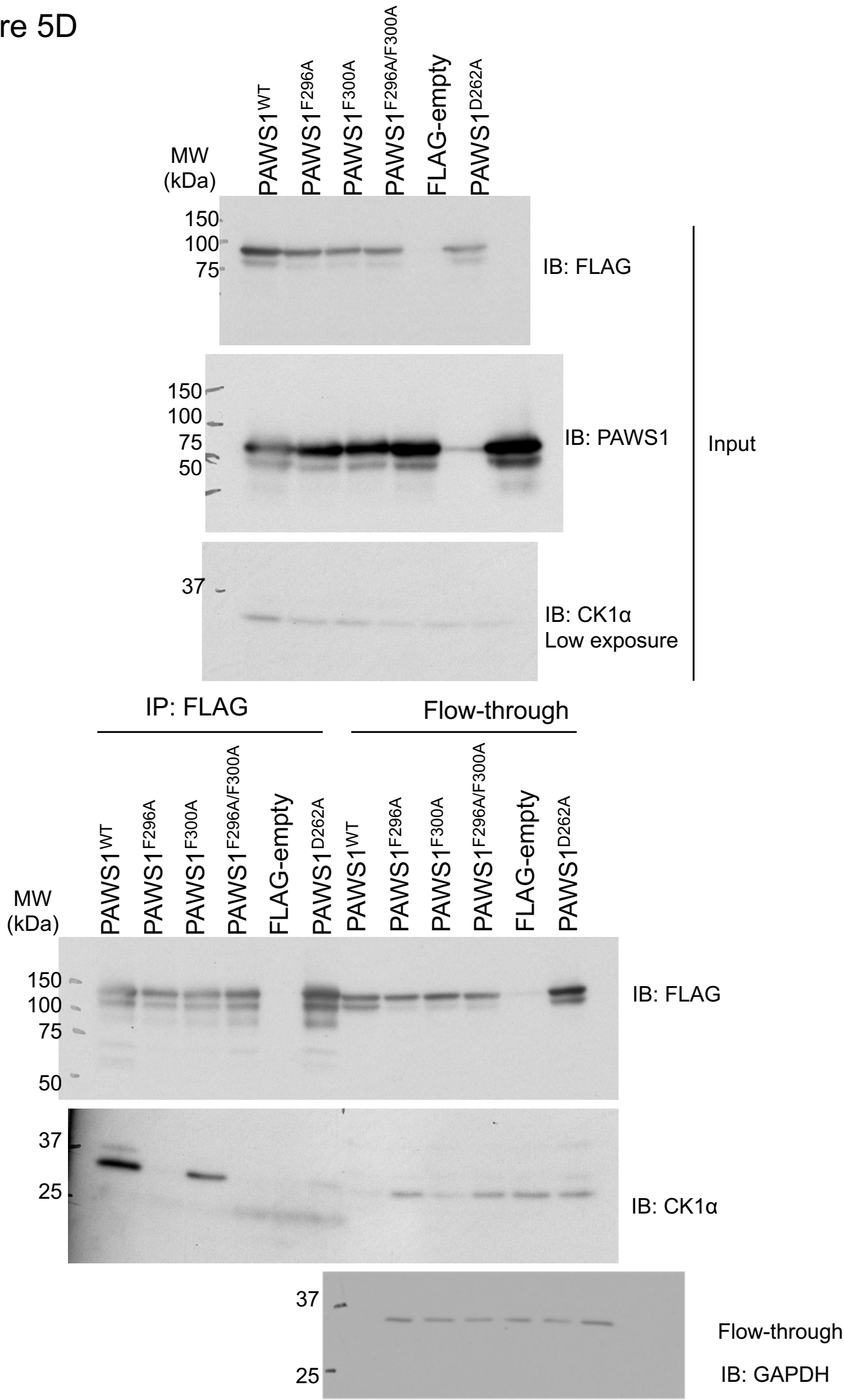

Figure 5E

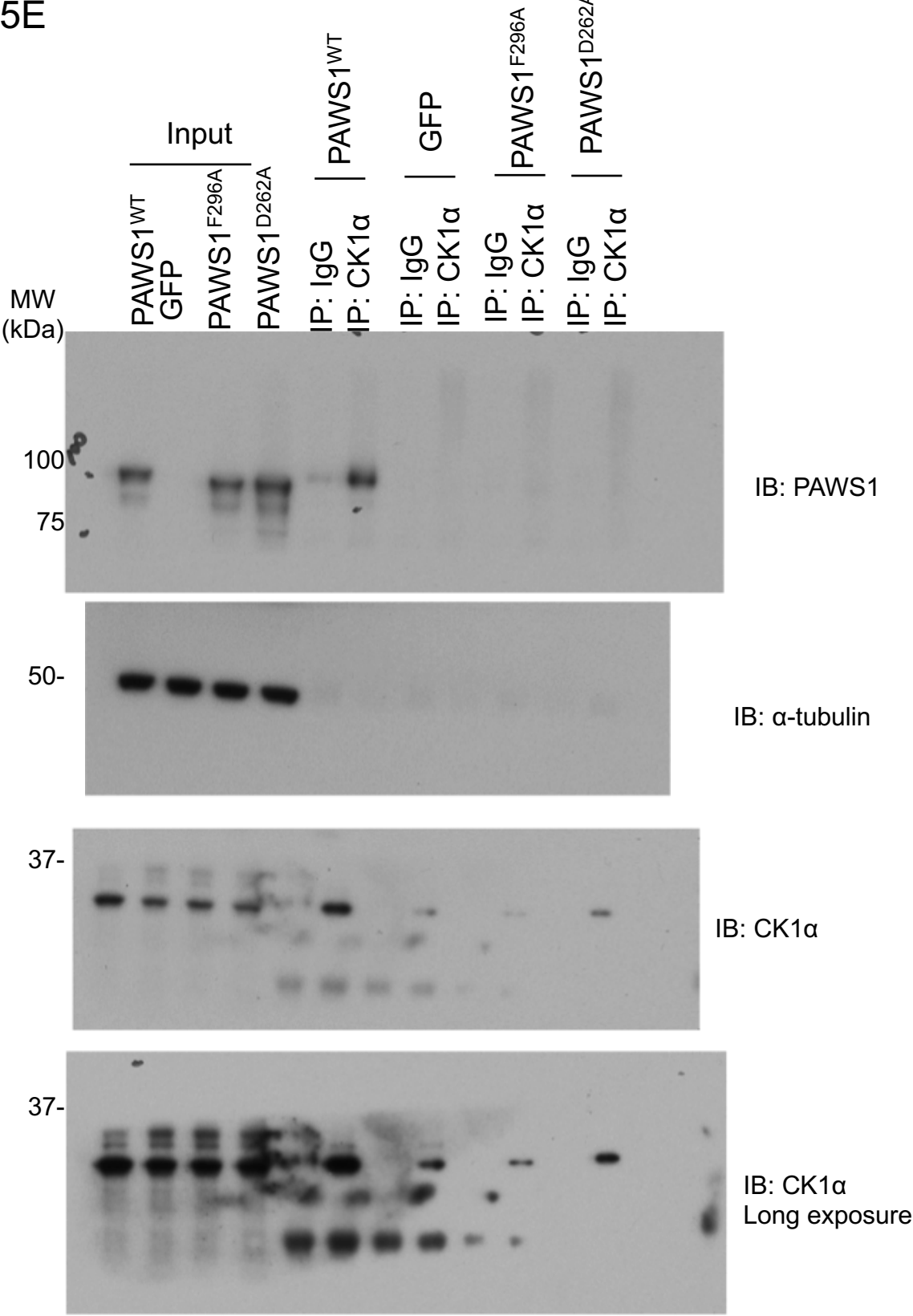

**Fig 5F**

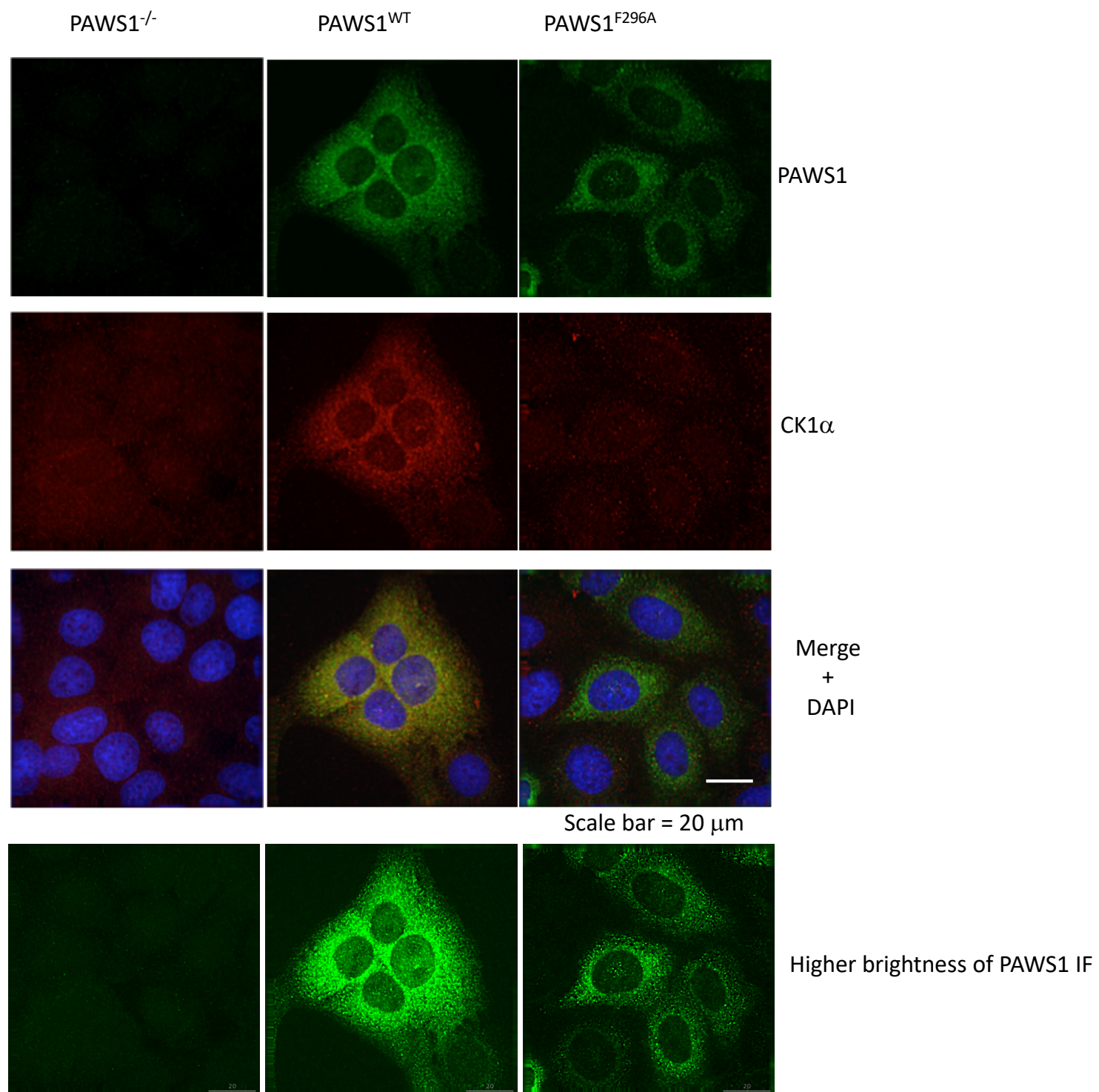

Supplement: Supplementary file 6 — Source Data for Figure 5 [file EMBR-19-e44807-s004.pdf]

Figure 6A

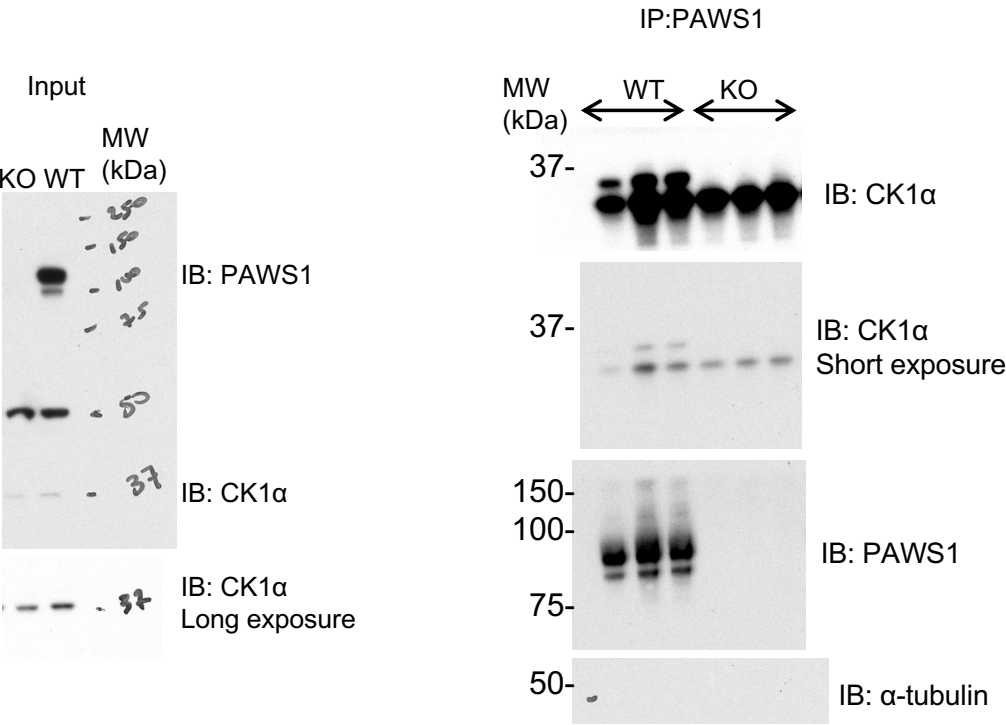

Figure 6

6C

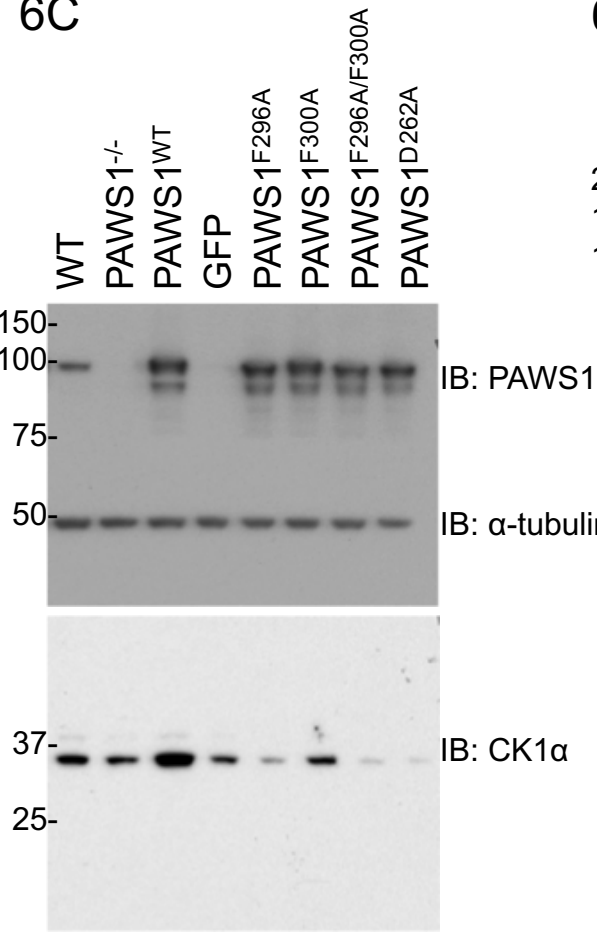

6D

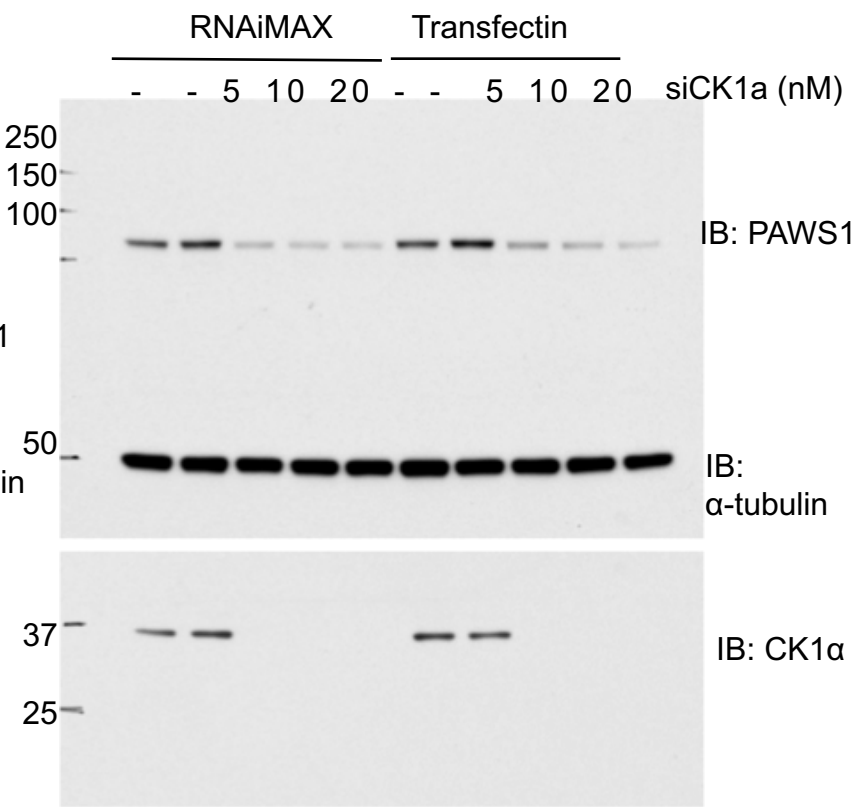

6E

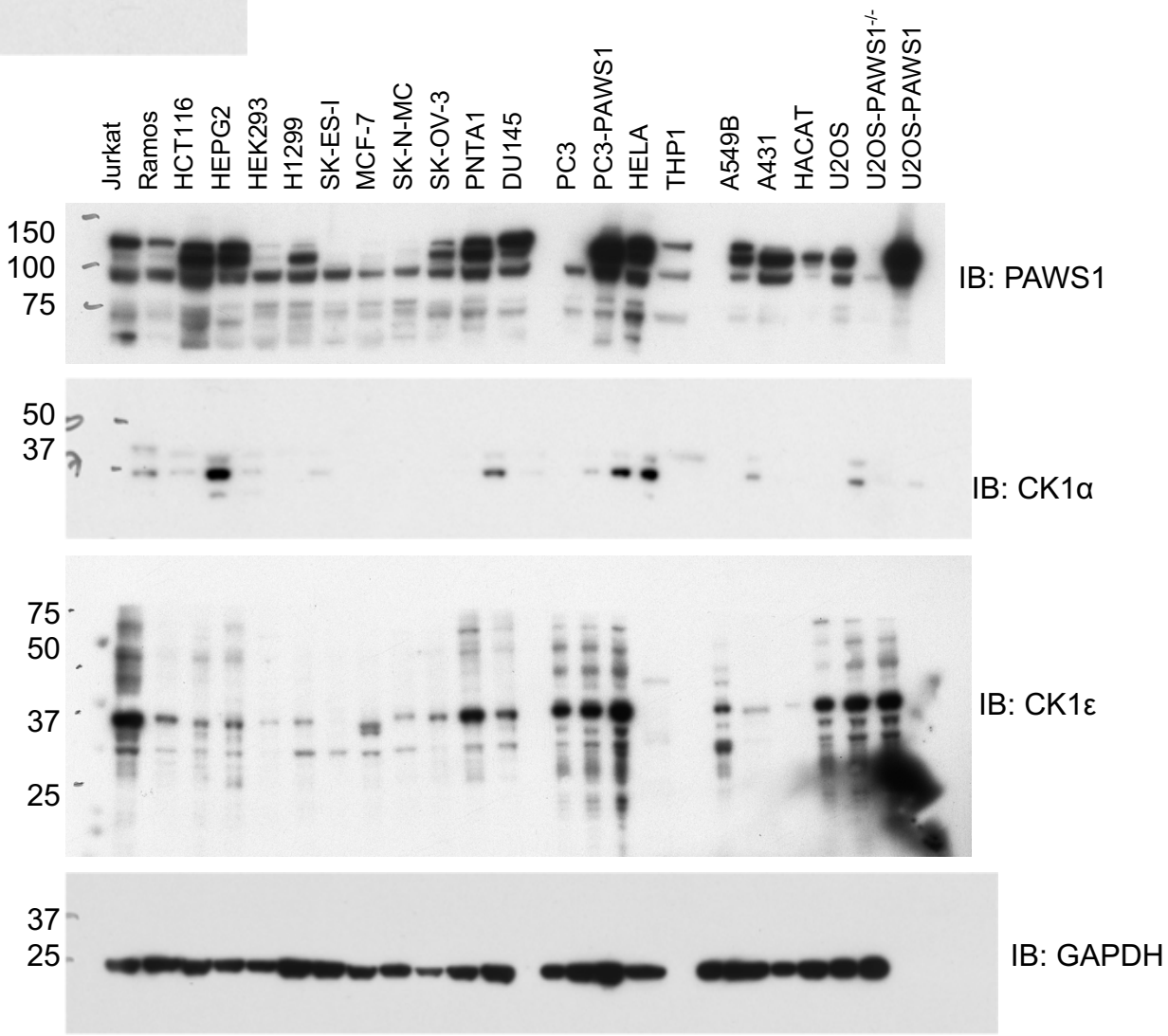

Supplement: Supplementary file 7 — Source Data for Figure 6 [file EMBR-19-e44807-s005.pdf]

Figure 7F

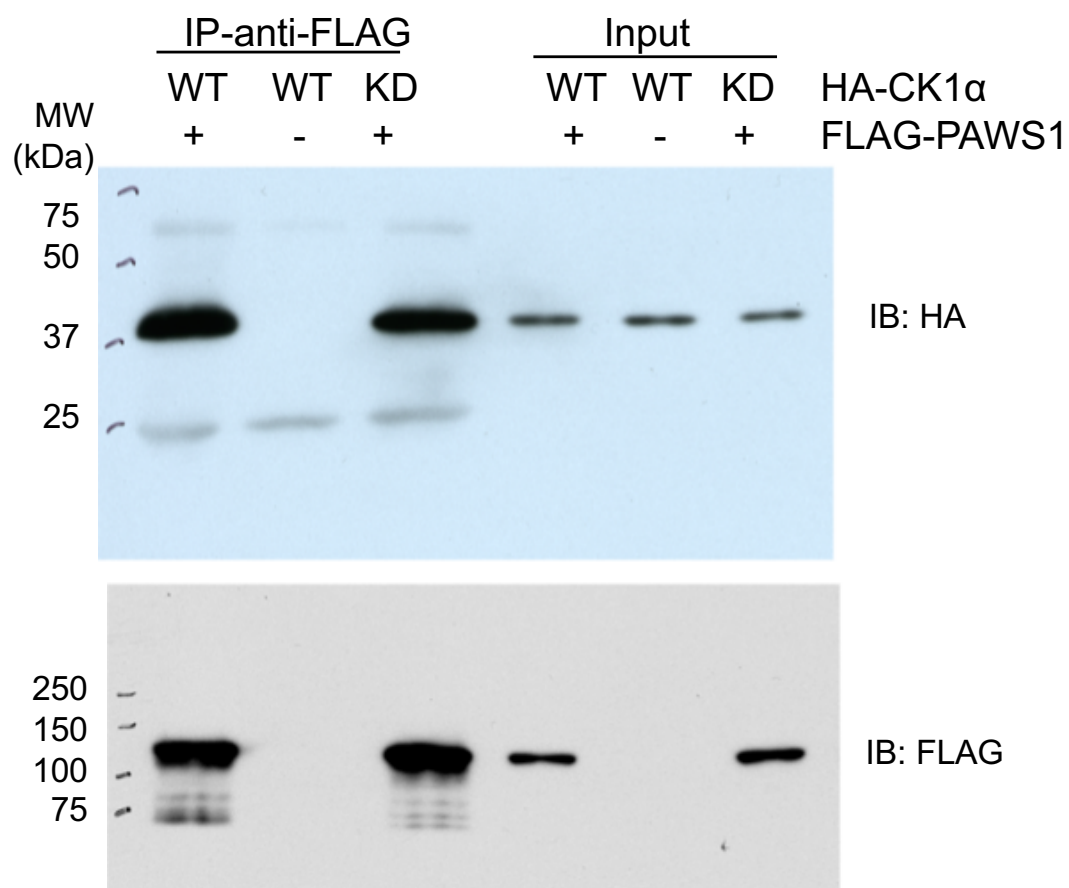

Supplement: Supplementary file 8 — Source Data for Figure 7 [file EMBR-19-e44807-s006.pdf]

Figure 8

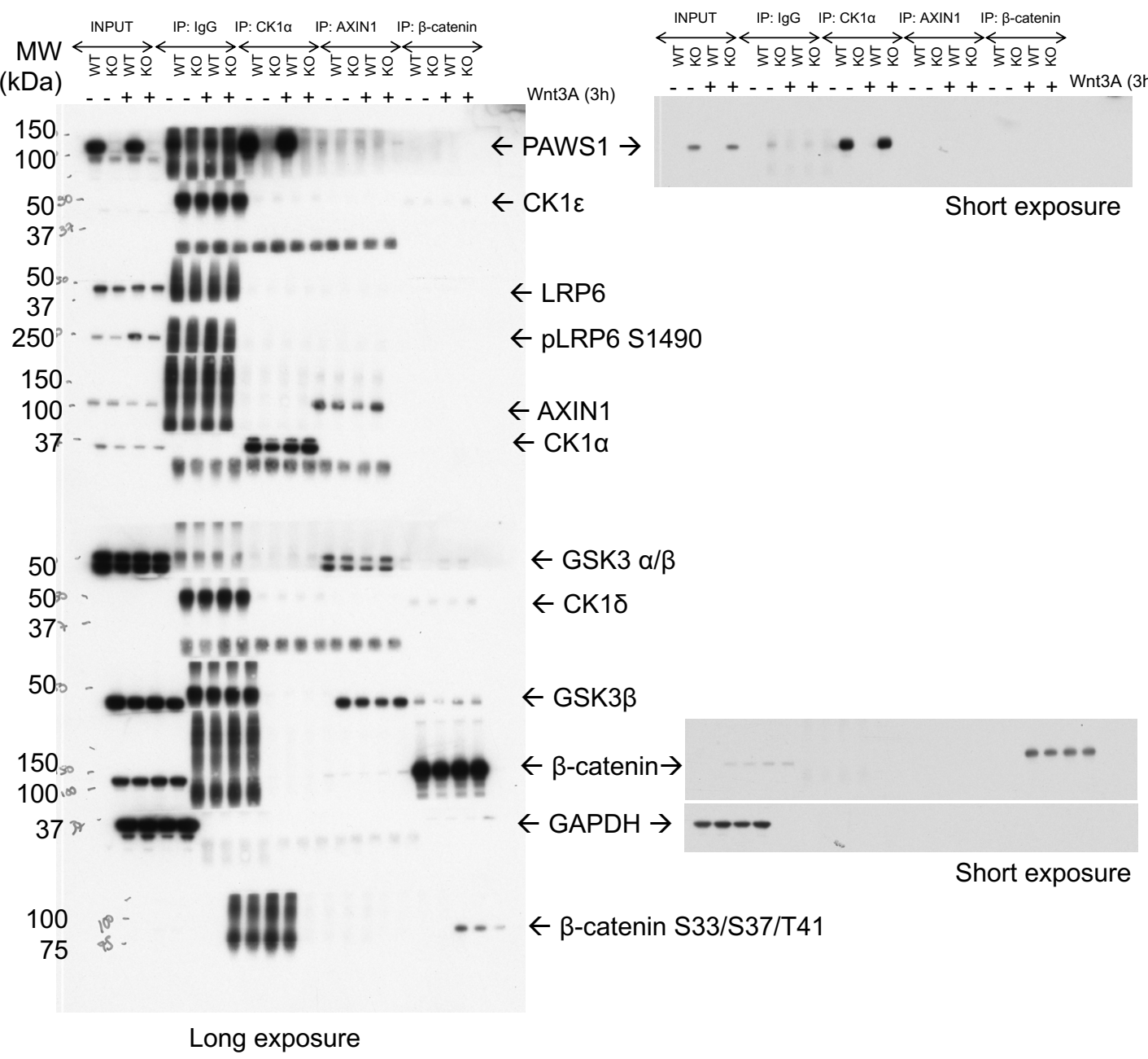

Supplement: Supplementary file 9 — Source Data for Figure 8 [file EMBR-19-e44807-s007.pdf]

Figure 9A

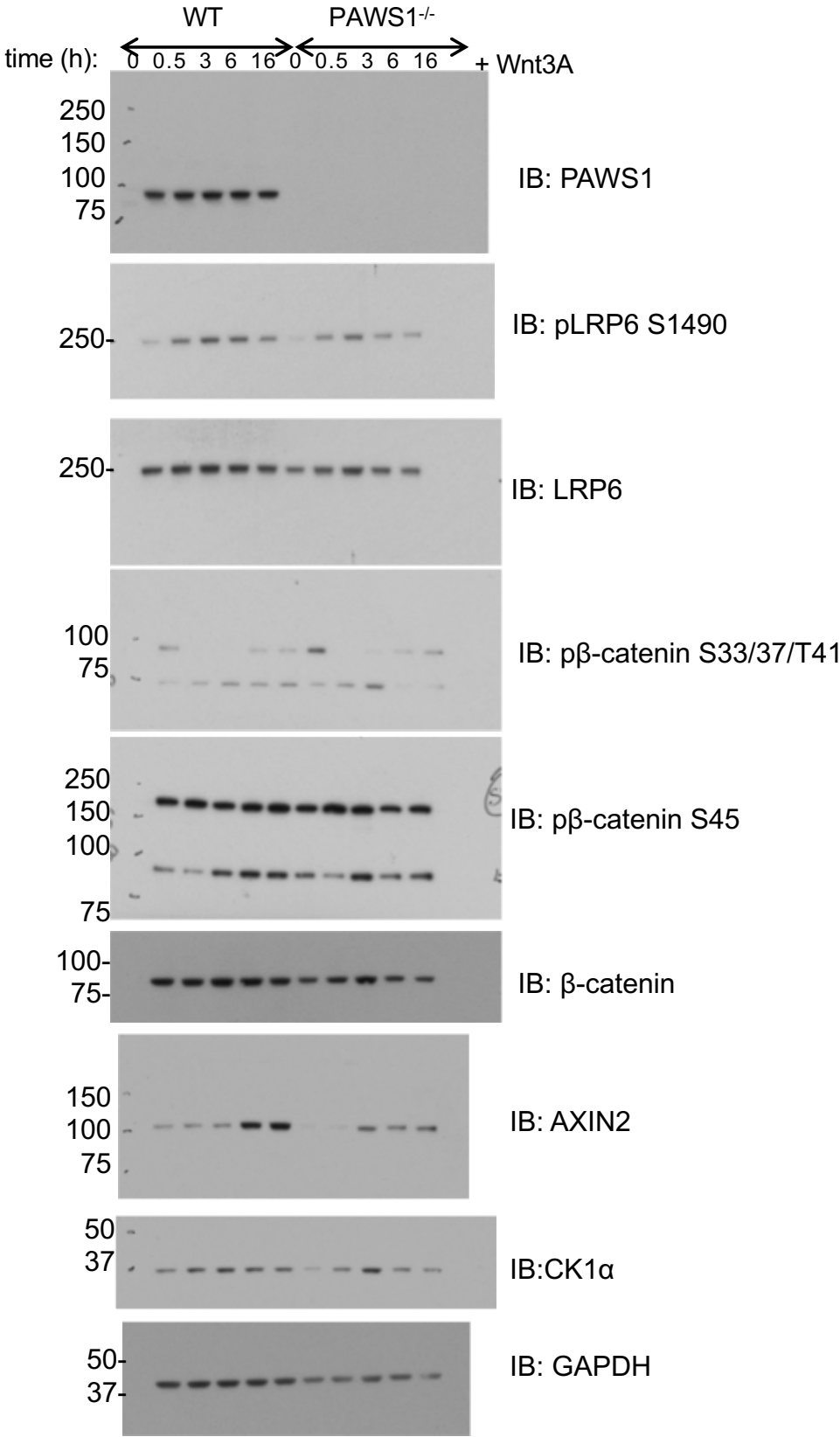

Figure 9B

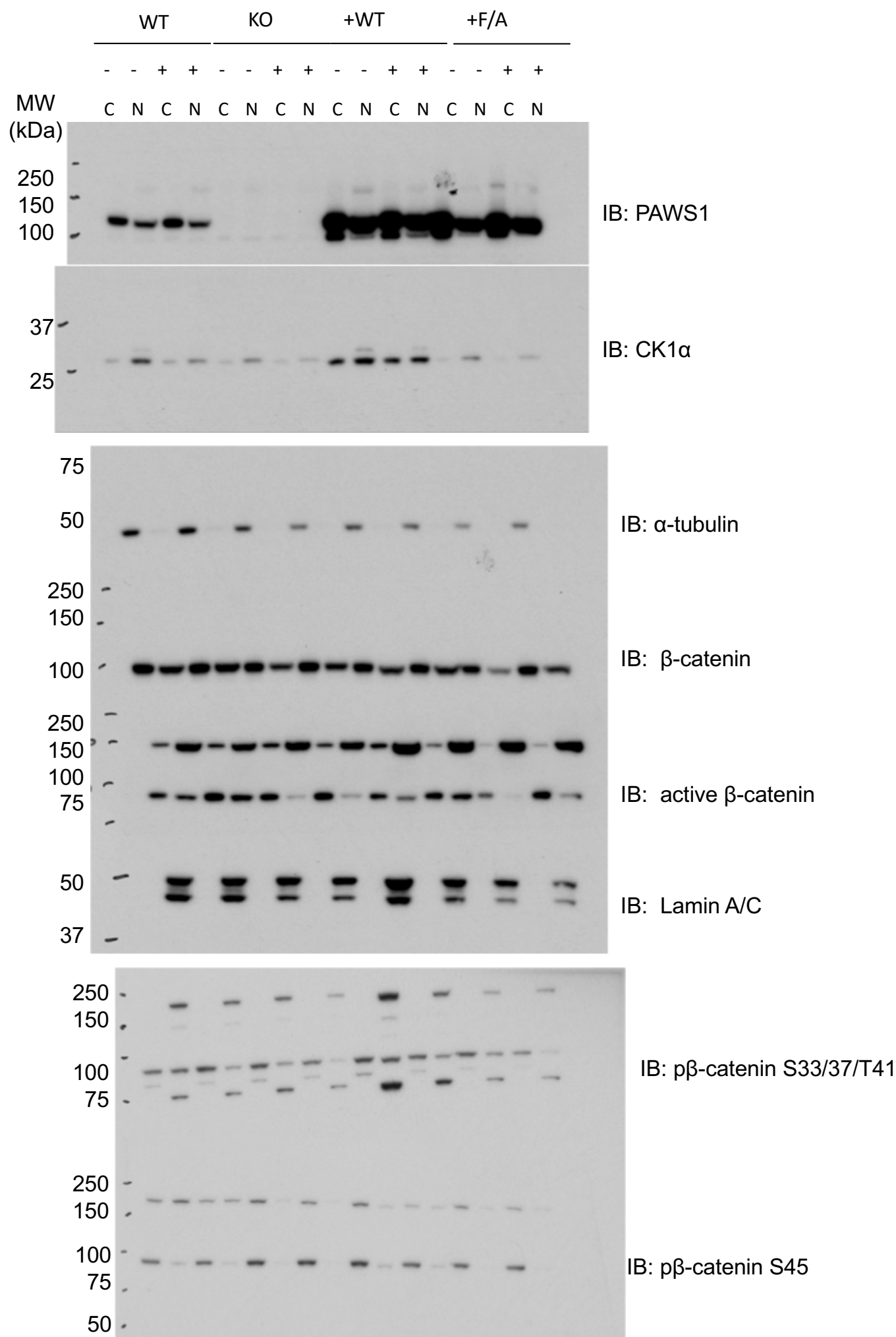

Supplement: Supplementary file 10 — Source Data for Figure 9 [file EMBR-19-e44807-s008.pdf]
