# Supplementary material for: PAWS1 controls Wnt signalling through association with casein kinase 1α
Source: EMBO Rep. 2018 Mar 7;19(4):e44807. doi: 10.15252/embr.201744807 (PMC5891436; doi:10.15252/embr.201744807)
Supplement: Supplementary file 5 — Source Data for Figure 3 [file EMBR-19-e44807-s003.pdf]

Figure 3A

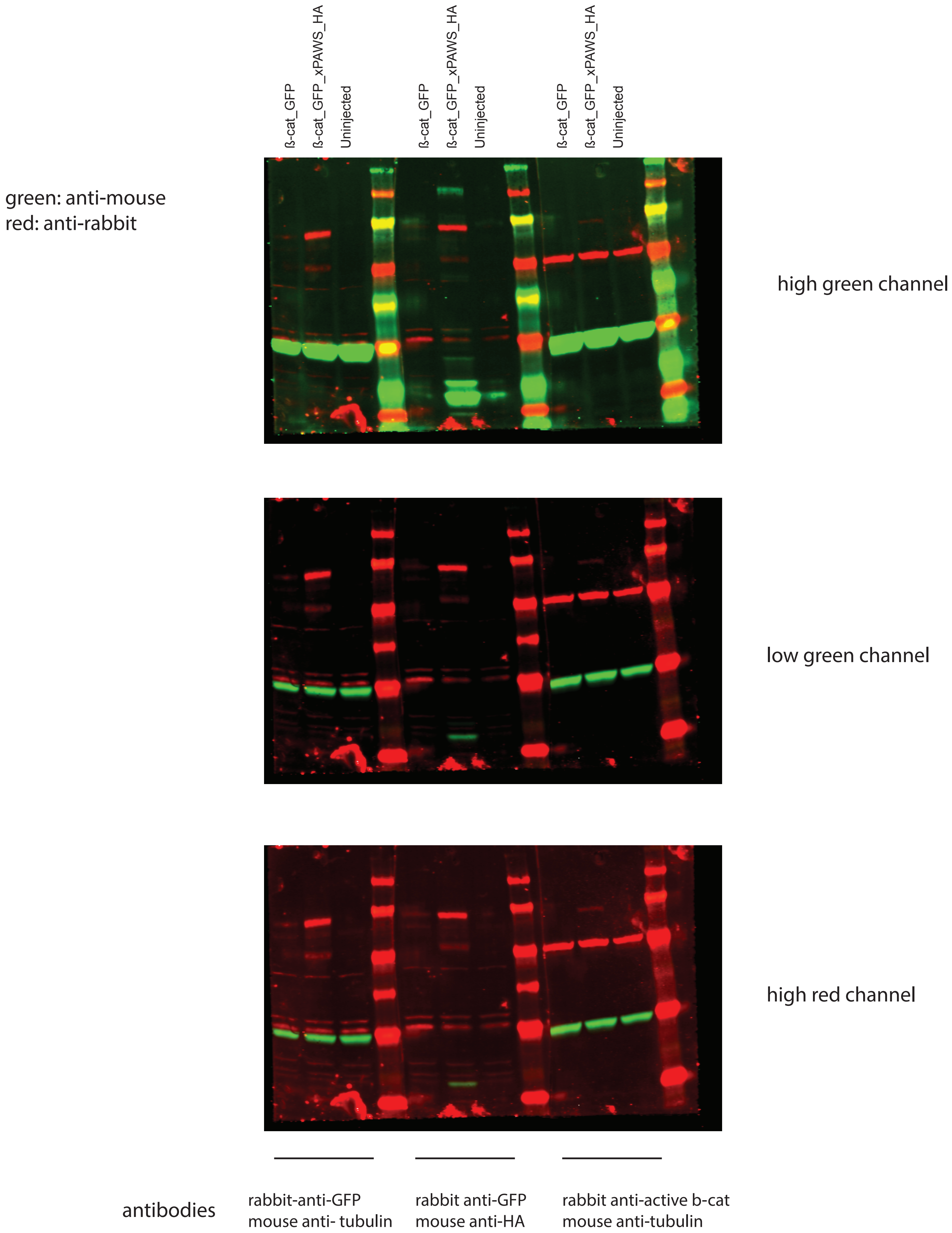

ß-cat_GFP
ß-cat_GFP_xPAWS_HA
Uninjected
ß-cat_GFP
ß-cat_GFP_xPAWS_HA
Uninjected
ß-cat_GFP
ß-cat_GFP_xPAWS_HA
Uninjected
green: anti-mouse
red: anti-rabbit
high green channel
low green channel
high red channel
antibodies
rabbit-anti-GFP
mouse anti- tubulin
rabbit anti-GFP
mouse anti-HA
rabbit anti-active b-cat
mouse anti-tubulin

Figure 3B

MT_PAWS1 | uninjected | PAWS1_mCherryHA | uninjected | MT_PAWS1 | uninjected | PAWS1_mCherryHA | uninjected

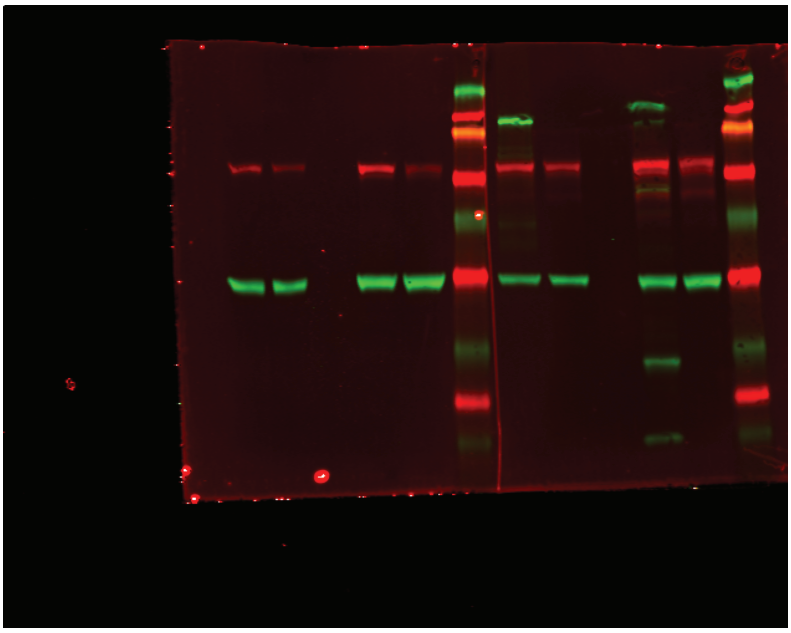

antibodies:

rabbit-anti-active b-cat
mouse-anti-tubulin

rabbit-anti-b-cat
mouse-anti-tubulin
mouse-anti-HA
mouse-anti-MT

anti-rabbit: red channel
anti-mouse: green channel

Fig 3E

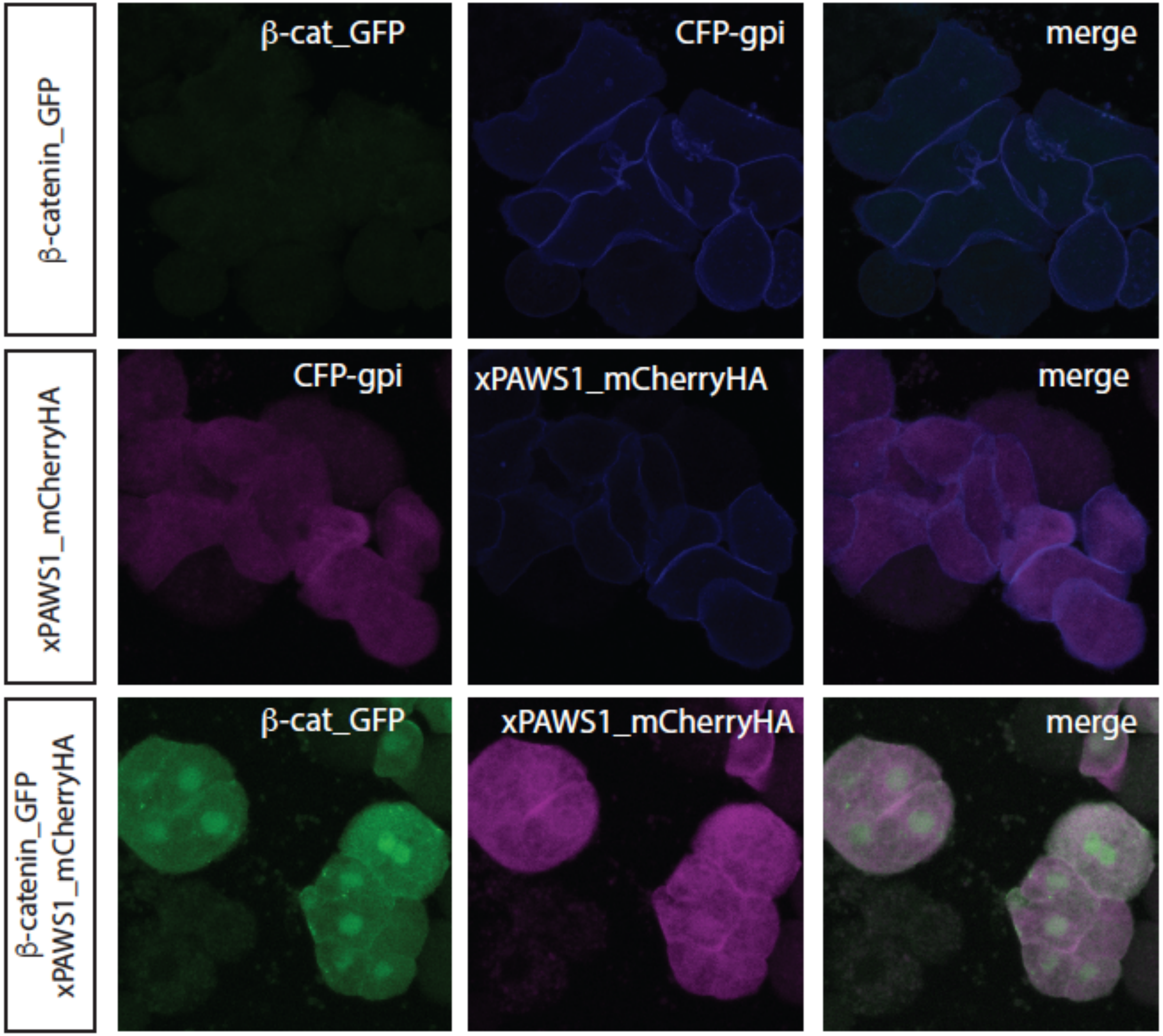
β-catenin_GFP
xPAWS1_mCherryHA
β-catenin_GFP xPAWS1_mCherryHA
β-cat_GFP
CFP-gpi
merge
CFP-gpi
xPAWS1_mCherryHA
merge
β-cat_GFP
xPAWS1_mCherryHA
merge
